# Supplementary material for: Life history and past demography maintain genetic structure, outcrossing rate, contemporary pollen gene flow of an understory herb in a highly fragmented rainforest
Source: PeerJ. 2016 Dec 22;4:e2764. doi: 10.7717/peerj.2764 (PMC5183091; doi:10.7717/peerj.2764)
Supplement: Table S1 [file peerj-04-2764-s001.docx]

Geographic coordinates for the studied populations of *A. aurantiaca* at Los Tuxtlas, México.

|  | Population | Name | Altitude | Coordinates | |
| --- | --- | --- | --- | --- | --- |
|  |  |  | masl | Latitude | longitude |
| Small | 1SM | Cola Pescado | 108 | 18.663183° | -95.145750° |
|  | 2SM | F1 | 82 | 18.577461° | -95.068416° |
|  | 3SM | Sta. Rosa | 82 | 18.549040° | -95.055235° |
|  | 4SM | San Pedro | 104 | \| 18.630437° \| \| --- \| | -95.110194° |
|  | 5SM | Playa | 94 | 18.588617° | -95.055900° |
| Medium | 6Med | Bambú | 538 | 18.610050° | -95.138717° |
|  | 7Med | Ruiz Cortines | 93 | 18.609440° | -95.096811° |
|  | 8Med | Borrego | 180 | 18.618656° | -95.085541° |
| Large | 9Lrg | Selva2 | 318 | 18.587717° | -95.100184° |
|  | 10Lrg | Selva1 | 100 | 18.585885° | -95.076820° |
|  | 11Lrg | Zacatal | 213 | 18.578804° | -95.089628° |
|  | 12Lrg | Vigia | 454 | 18.578114° | -95.080036° |
