# Supplementary material for: Life history and past demography maintain genetic structure, outcrossing rate, contemporary pollen gene flow of an understory herb in a highly fragmented rainforest
Source: PeerJ. 2016 Dec 22;4:e2764. doi: 10.7717/peerj.2764 (PMC5183091; doi:10.7717/peerj.2764)
Supplement: Table S2 [file peerj-04-2764-s002.docx]

Microsatellite loci of *Aphelandra aurantiaca*

| Locus name | Motif repeat | Primer sequences | Allele size | GeneBank  Accession no. |
| --- | --- | --- | --- | --- |
| 5490 | (ATGC)_24_ | F: GGTGTACGTAGCCCACAACG  R:TGAAGAAGTTGTTCCAAGGTACG | 174-184 | SRR1816884 |
| 0432 | (AGCC)_24_ | F:AGGCTGAAGAGATTTGCAGG  R:AAGACAGGCTGATGCAGTCG | 113-124 | SRR1816885 |
| 4343 | (ATT)_27_ | F: TGTAAAGGAAAGTTGAAGAAATAAGGG  R: TGATTCGTTGGAGACACATGC | 150-172 | SRR1817142 |
| 1233 | (AT)_22_ | F:GTTGCATTTGAGGCATGAGG  R:TGTAATTGAACTAGGTCTTGTACTCGC | 116-126 | SRR1514097 |
| 4914 | (AT)_22_ | F: AGGAATTGTCCGGTCTTCCC  R:CCGGCTGATTCTGCTTCC | 130-152 | SRR1817143 |
| 1810 | (AC)_26_ | F:TGGCACTTATAGCCACATCCC  R:GAACCAGTGTTGCGTGTCC | 194-207 | SRR1817168 |
| 4883 | (TC)_30_ | F:GATGGAGGCAGTGGAGATAGC  R:GCAGAATCTTCTGGAACCACC | 206-229 | SRR1817184 |
| 5250 | (TC)_28_ | F:TTCCTTCTTGTTGTTATTCTTGGC  R:GGAACAAAGAGTCATGATTGAAGC | 208-293 | SRR1817169 |
| 5441 | (TC)_30_ | F:CAAAGACCTGTAATAGATATAAGGAAGCC  R:AACTTAATGGACCATGTCGGC | 200-300 | SRR1817260 |
| 1808 | (AGT)_30_ | F:TGCGTGTCTTTGTTGTACTATCTGG  R:AATGCTCAAGGCATGCACC | 294-318 | SRR1817198 |
| 4536 | (TGC)_21_ | F: AAGAATTGTAATCCTTGAAAGCCC  R:GGAAATTTATATGGAATGCCGC | 187-193 | SRR1817191 |
