# Supplementary material for: Life history and past demography maintain genetic structure, outcrossing rate, contemporary pollen gene flow of an understory herb in a highly fragmented rainforest
Source: PeerJ. 2016 Dec 22;4:e2764. doi: 10.7717/peerj.2764 (PMC5183091; doi:10.7717/peerj.2764)
Supplement: Table S3 [file peerj-04-2764-s003.docx]

Priors parameters distribution of *A. aurantiaca* used in DIYABC and posterior parameters estimated for Scenario 4.

| Prior parameters |  | Prior distribution | Posterior parameters | | | | |
| --- | --- | --- | --- | --- | --- | --- | --- |
|  |  |  | Mean | Median | Mode | Quantile2.5% | Quantile97.5% |
| Population size assoc with expansion | *Ns* | UN 1000-100000 | 66100 | 68600 | 96000 | 21000 | 98500 |
| Population size assoc with bottleneck | *Nb* | UN 10-10000 | 1810 | 1180 | 482 | 64.2 | 6990 |
| Time since the expansion, years before present | *t* | UN 10-10000 | 2690 | 2030 | 712 | 254 | 8370 |

UN: Uniform distribution, with minimum and maximum values. We consider a generation time of approximately 1 year for *A. aurantica*
