# Supplementary material for: Life history and past demography maintain genetic structure, outcrossing rate, contemporary pollen gene flow of an understory herb in a highly fragmented rainforest
Source: PeerJ. 2016 Dec 22;4:e2764. doi: 10.7717/peerj.2764 (PMC5183091; doi:10.7717/peerj.2764)
Supplement: Table S4 — TPM: two-phase model of mutation; SMM: step-wise mutation model. *P < 0.05 indicates significance of Wilcoxon’s signed –rank test. [file peerj-04-2764-s004.doc]

Mean geographic distance (km, in lower diagonal) and Conditional genetic distances (cGD, in upper diagonal) between populations of *A. aurantiaca*.

|  |  | SMALL | | | | | MEDIUM | | | LARGE | | | |
| --- | --- | --- | --- | --- | --- | --- | --- | --- | --- | --- | --- | --- | --- |
|  | Pop | 1SM | 2SM | 3SM | 4SM | 5SM | 6Med | 7Med | 8Med | 9Lrg | 10Lrg | 11Lrg | 12Lrg |
| SMALL | 1SM | **0** | 4.73 | 8.27 | 10.3 | 9.5 | 13.9 | 10.8 | 5.31 | 13.3 | 10.5 | 5.00 | 9.36 |
|  | 2SM | 12.56 | **0** | 11.9 | 5.65 | 9.07 | 12.4 | 11.4 | 10.0 | 8.57 | 11.5 | 4.55 | 4.63 |
|  | 3SM | 15.91 | 3.46 | **0** | 6.59 | 15.5 | 11.9 | 7.40 | 12.3 | 11.2 | 7.77 | 11.7 | 7.32 |
|  | 4SM | 5.28 | 7.3 | 10.72 | **0** | 14.7 | 10.2 | 5.78 | 5.77 | 14.1 | 5.91 | 10.2 | 10.2 |
|  | 5SM | 12.59 | 1.76 | 4.36 | 7.36 | **0** | 4.43 | 8.94 | 14.4 | 4.24 | 9.57 | 4.52 | 8.18 |
| MEDIUM | 6Med | 5.95 | 8.29 | 11.16 | 3.81 | 9.11 | **0** | 4.50 | 10.0 | 3.89 | 5.13 | 5.13 | 8.95 |
|  | 7Med | 7.91 | 4.65 | 7.98 | 2.74 | 4.87 | 4.5 | **0** | 5.53 | 8.40 | 9.64 | 13.4 | 12.3 |
|  | 8Med | 8.1 | 4.89 | 8.36 | 2.9 | 4.56 | 5.74 | 1.56 | **0** | 13.9 | 5.22 | 10.32 | 14.68 |
| LARGE | 9Lrg | 6.44 | 3.61 | 6.42 | 4.83 | 4.7 | 4.81 | 2.45 | 3.76 | **0** | 9.02 | 8.34 | 3.94 |
|  | 10Lrg | 11.3 | 1.31 | 4.75 | 6.07 | 2.25 | 7.12 | 3.33 | 3.73 | 2.47 | **0** | 14.0 | 12.9 |
|  | 11Lrg | 4.99 | 2.26 | 4.99 | 6.11 | 3.73 | 6.29 | 3.37 | 4.32 | 1.44 | 1.55 | **0** | 4.39 |
|  | 12Lrg | 11.72 | 1.22 | 4.41 | 6.66 | 2.8 | 7.16 | 3.86 | 4.54 | 2.38 | 0.92 | 1 | **0** |
