# Supplementary material for: Life history and past demography maintain genetic structure, outcrossing rate, contemporary pollen gene flow of an understory herb in a highly fragmented rainforest
Source: PeerJ. 2016 Dec 22;4:e2764. doi: 10.7717/peerj.2764 (PMC5183091; doi:10.7717/peerj.2764)
Supplement: Table S5 — Bold numbers indicate membership probabilities for individuals in their home population. [file peerj-04-2764-s005.doc]

Tests of population bottleneck of *A. aurantiaca* in different fragment size.

|  | Bottleneck | | | | | | | |
| --- | --- | --- | --- | --- | --- | --- | --- | --- |
|  | TPM | | | | SMM | | | |
| fragment | *He excess (eq.)* | *Ho excess* | *H deficit* | *P* | *He excess (eq.)* | *Ho excess* | *H deficit* | *P* |
| Small | 6.45 | 1 | 10 | 0.002* | 6.43 | 1 | 10 | 0.002* |
| Medium | 6.49 | 2 | 9 | 0.004* | 6.47 | 2 | 9 | 0.009* |
| Large | 6.48 | 0 | 11 | 0.004* | 6.53 | 0 | 11 | 0.004* |

Tests were based on heterozygosity excess. TPM: two-phase model of mutation; SMM: step-wise mutation model. **P*< 0.05 indicates significance of Wilcoxon’s signed –rank test.
