# Supplementary material for: Life history and past demography maintain genetic structure, outcrossing rate, contemporary pollen gene flow of an understory herb in a highly fragmented rainforest
Source: PeerJ. 2016 Dec 22;4:e2764. doi: 10.7717/peerj.2764 (PMC5183091; doi:10.7717/peerj.2764)
Supplement: Table S6 — Footnote: Resident fragment refers to the fragment size an individual was found at, fragment source indicates the most likely origin. L was estimated as L= -LOG (Lhome), where L is the ratio of the likelihood computed from the population where the individual was sampled (Lhome). Fifteen migrants were identified across the different fragment sizes. Average membership probability to resident population was estimated in DAPC. [file peerj-04-2764-s006.docx]

Membership probabilities of *A. aurantiaca* to their populations of sampling extracted from DAPC. Bold numbers indicate membership probabilities for individuals in their home population.

|  | 1SM | 2SM | 3SM | 4SM | 5SM | 6Med | 7Med | 8Med | 9Lrg | 10Lrg | 11Lrg | 12Lrg |
| --- | --- | --- | --- | --- | --- | --- | --- | --- | --- | --- | --- | --- |
| 1SM | **0.644** | *0.070* | 5E-4 | 2E-4 | 0.031 | 0.019 | 0.009 | *0.053* | 0.034 | *0.085* | 0.021 | 0.028 |
| 2SM | *0.057* | **0.633** | 4.3E-7 | 0.007 | 0.018 | 0.018 | 0.075 | 0.010 | 0.049 | 0.043 | *0.065* | 0.018 |
| 3SM | 0.001 | 0.003 | **0.778** | *0.243* | 0.007 | 0.009 | *0.098* | 0.003 | 0.011 | 0.035 | 0.019 | 0.007 |
| 4SM | 3E-4 | 0.034 | *0.040* | **0.658** | 0.020 | 0.013 | *0.083* | 0.008 | 0.045 | *0.061* | 0.016 | 0.016 |
| 5SM | 0.033 | 0.012 | 9.6E-7 | 0.034 | **0.537** | *0.062* | 0.035 | 0.023 | *0.109* | 0.020 | *0.077* | 0.054 |
| 6Med | 0.012 | 0.003 | 1.8E-5 | 0.001 | 0.092 | **0.564** | *0.080* | 0.038 | 0.073 | 0.022 | *0.075* | 0.034 |
| 7Med | 0.029 | 0.049 | 8.3E-5 | 0.014 | 0.015 | *0.073* | **0.586** | 0.011 | 0.040 | 0.043 | 0.059 | *0.075* |
| 8Med | *0.058* | 0.035 | 1.6E-6 | 0.007 | 0.032 | 0.013 | 0.026 | **0.592** | *0.084* | *0.120* | 0.022 | 0.007 |
| 9Lrg | 0.025 | 0.028 | 1.6E-6 | 0.008 | *0.067* | *0.101* | 0.048 | *0.067* | **0.465** | *0.061* | 0.044 | *0.081* |
| 10Lrg | 0.004 | 0.012 | 8.2E-6 | 0.010 | 0.045 | 0.055 | 0.013 | *0.090* | *0.079* | **0.587** | 0.057 | 0.041 |
| 11Lrg | 0.022 | *0.056* | 1.1E-6 | 0.007 | 0.049 | *0.063* | 0.040 | 0.019 | *0.076* | 0.035 | **0.555** | *0.072* |
| 12Lrg | 0.026 | *0.086* | 3.2E-7 | 0.015 | 0.043 | 0.032 | 0.019 | 0.009 | *0.090* | 0.025 | *0.075* | **0.574** |
