# Supplementary figures and images for: Life history and past demography maintain genetic structure, outcrossing rate, contemporary pollen gene flow of an understory herb in a highly fragmented rainforest
Source: PeerJ. 2016 Dec 22;4:e2764. doi: 10.7717/peerj.2764 (PMC5183091; doi:10.7717/peerj.2764)

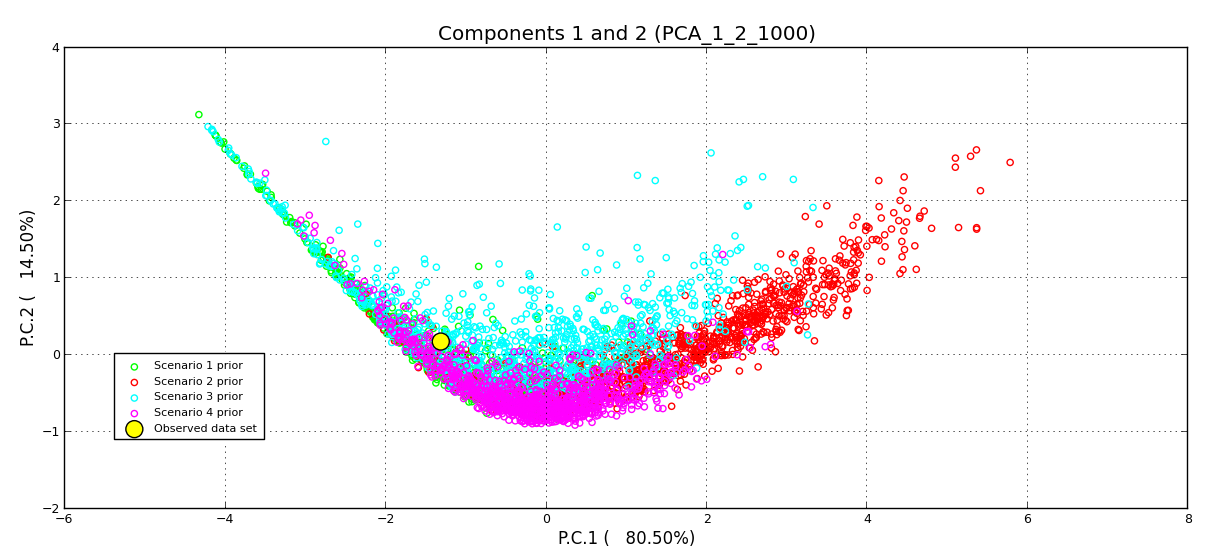

Supplement: Figure S1 — Scenario 4 fits the observed data best. [file peerj-04-2764-s007.png]

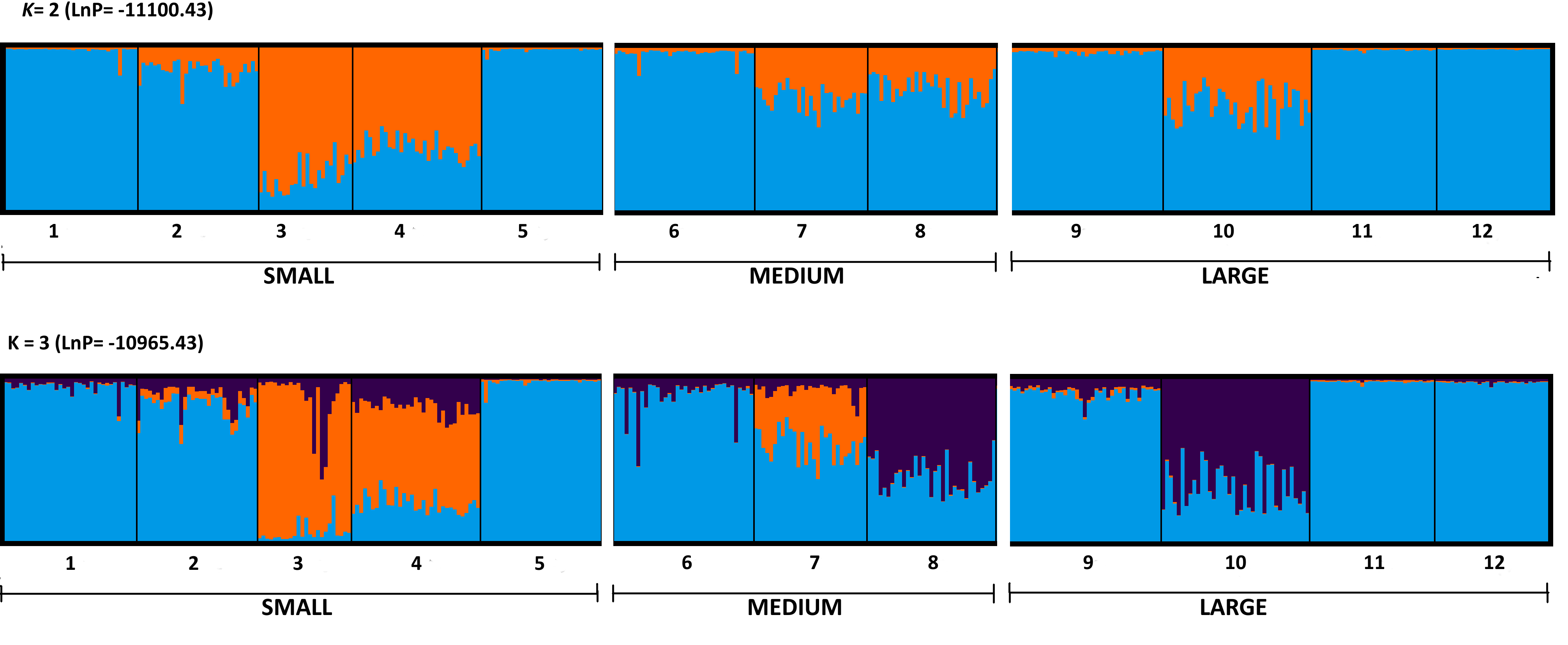

Supplement: Figure S2 — K = 2 and K = 3 were detected by Evanno, Regnaut & Goudet (2005) method of optimal number of genetic clusters. Each bar represents an individual and its proportional memebership to clusters. Populations are ordered from small to large fragments. [file peerj-04-2764-s008.png]

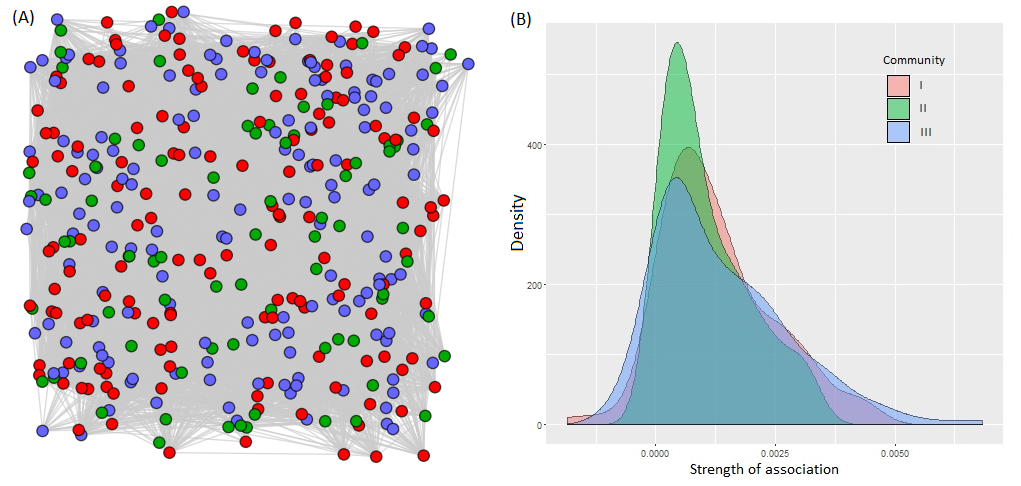

Supplement: Figure S3 — (A) Community detection on Aphelandra aurantiaca network with medium threshold (0.245), using Fast Greedy and geographic coordinates algorithm implemented in NetStruct. Each node represents an individual, with colors representing the three community assigned. (B) SAD Analysis for the network, show the distributions of the SA values for each community. [file peerj-04-2764-s009.png]

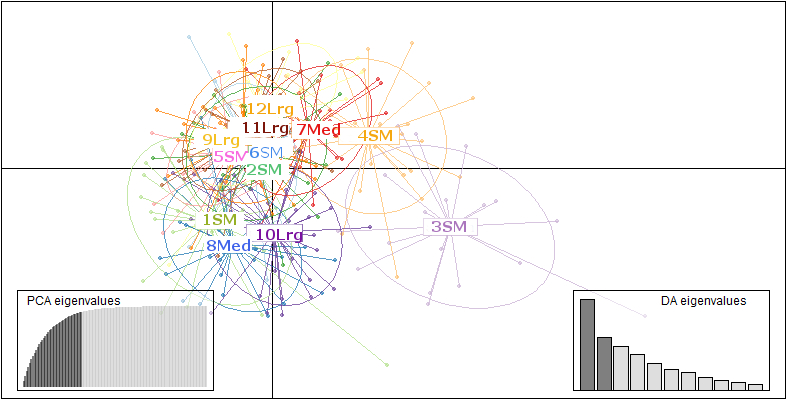

Supplement: Figure S4 [file peerj-04-2764-s010.jpg]
