# Supplementary material for: Life history and past demography maintain genetic structure, outcrossing rate, contemporary pollen gene flow of an understory herb in a highly fragmented rainforest
Source: PeerJ. 2016 Dec 22;4:e2764. doi: 10.7717/peerj.2764 (PMC5183091; doi:10.7717/peerj.2764)
Supplement: Data S1 [file peerj-04-2764-s011.pdf]

12 populations, 11 microsatellite			Primer 1		Primer 2	
Fragment size	Individual Num.	Population	5409-1	5409-2	432-1	432-2
SMALL	1	1 SM	180	184	93	103
SMALL	2	1 SM	180	184	96	103
SMALL	3	1 SM	182	184	103	103
SMALL	4	1 SM	180	180	99	99
SMALL	5	1 SM	184	184	99	103
SMALL	6	1 SM	182	184	93	99
SMALL	7	1 SM	180	184	96	99
SMALL	8	1 SM	180	180	96	99
SMALL	9	1 SM	180	184	99	99
SMALL	10	1 SM	184	184	103	103
SMALL	11	1 SM	184	184	99	99
SMALL	12	1 SM	184	184	99	99
SMALL	13	1 SM	180	184	99	99
SMALL	14	1 SM	180	184	99	99
SMALL	15	1 SM	180	180	96	99
SMALL	16	1 SM	180	180	93	96
SMALL	17	1 SM	180	180	93	93
SMALL	18	1 SM	180	180	99	99
SMALL	19	1 SM	184	184	103	103
SMALL	20	1 SM	182	182	99	103
SMALL	21	1 SM	180	184	99	99
SMALL	22	1 SM	180	180	99	99
SMALL	23	1 SM	182	184	103	103
SMALL	24	1 SM	184	184	99	99
SMALL	25	1 SM	180	184	99	99
SMALL	26	1 SM	180	184	99	103
SMALL	27	1 SM	182	184	103	103
SMALL	28	1 SM	182	184	99	99
SMALL	29	1 SM	182	184	93	99
SMALL	30	1 SM	180	182	96	99
SMALL	31	1 SM	182	182	99	103
SMALL	32	1 SM	174	178	99	99
SMALL	33	1 SM	182	184	99	99
SMALL	34	1 SM	180	180	93	96
SMALL	1	2 SM	180	184	93	93
SMALL	2	2 SM	180	184	93	103
SMALL	3	2 SM	180	184	99	99
SMALL	4	2 SM	180	184	93	99
SMALL	5	2 SM	182	184	99	99
SMALL	6	2 SM	180	184	93	99
SMALL	7	2 SM	180	184	96	99
SMALL	8	2 SM	180	184	99	99
SMALL	9	2 SM	180	184	93	99
SMALL	10	2 SM	180	184	96	103
SMALL	11	2 SM	180	184	96	96
SMALL	12	2 SM	180	184	96	99
SMALL	13	2 SM	180	184	99	103

SMALL	14	2 SM	180	180	93	99
SMALL	15	2 SM	180	182	96	99
SMALL	16	2 SM	180	184	93	93
SMALL	17	2 SM	180	184	96	103
SMALL	18	2 SM	180	184	96	103
SMALL	19	2 SM	180	184	99	103
SMALL	20	2 SM	180	184	99	99
SMALL	21	2 SM	182	184	96	103
SMALL	22	2 SM	180	184	99	103
SMALL	23	2 SM	180	184	99	103
SMALL	24	2 SM	180	184	99	99
SMALL	25	2 SM	180	184	99	103
SMALL	26	2 SM	180	184	99	103
SMALL	27	2 SM	180	184	96	103
SMALL	28	2 SM	184	184	96	99
SMALL	29	2 SM	180	184	96	99
SMALL	30	2 SM	180	184	93	103
SMALL	31	2 SM	180	184	99	103
SMALL	1	3 SM	180	180	99	99
SMALL	2	3 SM	179	184	96	99
SMALL	3	3 SM	180	180	99	99
SMALL	4	3 SM	180	180	99	99
SMALL	5	3 SM	180	184	99	99
SMALL	6	3 SM	180	180	99	99
SMALL	7	3 SM	180	180	99	99
SMALL	8	3 SM	180	180	99	99
SMALL	9	3 SM	180	180	99	99
SMALL	10	3 SM	180	184	99	99
SMALL	11	3 SM	180	184	99	99
SMALL	12	3 SM	180	184	99	99
SMALL	13	3 SM	180	184	99	99
SMALL	14	3 SM	-9	-9	-9	-9
SMALL	15	3 SM	180	180	99	99
SMALL	16	3 SM	180	184	99	99
SMALL	17	3 SM	180	180	99	99
SMALL	18	3 SM	180	180	99	99
SMALL	19	3 SM	180	180	99	99
SMALL	20	3 SM	180	180	99	99
SMALL	21	3 SM	180	180	99	99
SMALL	22	3 SM	180	180	99	99
SMALL	23	3 SM	180	180	99	99
SMALL	24	3 SM	180	184	99	99
SMALL	1	4 SM	184	184	99	99
SMALL	2	4 SM	180	182	96	103
SMALL	3	4 SM	180	180	93	99
SMALL	4	4 SM	180	182	96	99
SMALL	5	4 SM	180	182	99	99
SMALL	6	4 SM	180	180	96	99
SMALL	7	4 SM	180	180	99	99

SMALL	8	4 SM	180	180	99	99
SMALL	9	4 SM	180	180	96	96
SMALL	10	4 SM	180	184	99	103
SMALL	11	4 SM	184	184	99	99
SMALL	12	4 SM	180	184	99	99
SMALL	13	4 SM	180	184	96	96
SMALL	14	4 SM	180	180	99	103
SMALL	15	4 SM	180	180	96	99
SMALL	16	4 SM	180	180	93	99
SMALL	17	4 SM	180	184	99	99
SMALL	18	4 SM	184	184	99	99
SMALL	19	4 SM	180	184	96	99
SMALL	20	4 SM	180	184	99	99
SMALL	21	4 SM	180	180	99	99
SMALL	22	4 SM	180	180	93	96
SMALL	23	4 SM	180	180	96	99
SMALL	24	4 SM	180	184	96	96
SMALL	25	4 SM	180	180	103	103
SMALL	26	4 SM	180	184	96	99
SMALL	27	4 SM	180	180	99	103
SMALL	28	4 SM	180	180	99	103
SMALL	29	4 SM	180	184	96	99
SMALL	30	4 SM	180	180	96	103
SMALL	31	4 SM	180	180	99	99
SMALL	32	4 SM	180	180	96	99
SMALL	33	4 SM	180	184	99	99
SMALL	1	5 SM	180	184	99	103
SMALL	2	5 SM	180	180	99	103
SMALL	3	5 SM	180	184	96	99
SMALL	4	5 SM	180	184	96	99
SMALL	5	5 SM	180	180	96	99
SMALL	6	5 SM	180	180	99	103
SMALL	7	5 SM	180	184	96	99
SMALL	8	5 SM	180	184	96	99
SMALL	9	5 SM	180	184	99	99
SMALL	10	5 SM	180	184	99	99
SMALL	11	5 SM	184	184	99	99
SMALL	12	5 SM	180	180	96	99
SMALL	13	5 SM	180	180	93	99
SMALL	14	5 SM	182	182	99	99
SMALL	15	5 SM	180	182	99	99
SMALL	16	5 SM	180	184	96	99
SMALL	17	5 SM	180	184	96	99
SMALL	18	5 SM	180	180	99	99
SMALL	19	5 SM	180	180	99	103
SMALL	20	5 SM	180	182	99	99
SMALL	21	5 SM	180	180	96	99
SMALL	22	5 SM	180	184	96	99
SMALL	23	5 SM	180	182	96	99

SMALL	24	5 SM	180	180	99	103
SMALL	25	5 SM	180	180	93	103
SMALL	26	5 SM	180	184	96	99
SMALL	27	5 SM	180	184	103	103
SMALL	28	5 SM	180	180	99	93
SMALL	29	5 SM	180	180	99	103
SMALL	30	5 SM	180	180	96	99
SMALL	31	5 SM	180	184	96	99
MEDIUM	1	6 Med	184	184	99	99
MEDIUM	2	6 Med	184	184	99	99
MEDIUM	3	6 Med	180	180	99	99
MEDIUM	4	6 Med	180	180	96	96
MEDIUM	5	6 Med	180	184	99	99
MEDIUM	6	6 Med	180	180	96	99
MEDIUM	7	6 Med	184	184	99	99
MEDIUM	8	6 Med	180	184	99	99
MEDIUM	9	6 Med	180	180	96	99
MEDIUM	10	6 Med	182	182	96	96
MEDIUM	11	6 Med	184	184	99	103
MEDIUM	12	6 Med	180	180	103	103
MEDIUM	13	6 Med	180	182	99	99
MEDIUM	14	6 Med	180	180	99	103
MEDIUM	15	6 Med	180	184	96	99
MEDIUM	16	6 Med	180	180	96	99
MEDIUM	17	6 Med	180	184	99	103
MEDIUM	18	6 Med	180	184	99	99
MEDIUM	19	6 Med	180	180	96	96
MEDIUM	20	6 Med	180	180	99	99
MEDIUM	21	6 Med	180	184	93	99
MEDIUM	22	6 Med	180	184	99	103
MEDIUM	23	6 Med	180	180	103	103
MEDIUM	24	6 Med	180	184	-9	-9
MEDIUM	25	6 Med	180	182	96	99
MEDIUM	26	6 Med	180	184	99	99
MEDIUM	27	6 Med	180	180	96	99
MEDIUM	28	6 Med	182	184	-9	-9
MEDIUM	29	6 Med	182	182	-9	-9
MEDIUM	30	6 Med	184	184	99	99
MEDIUM	31	6 Med	182	184	99	99
MEDIUM	32	6 Med	182	184	99	99
MEDIUM	33	6 Med	180	184	99	103
MEDIUM	34	6 Med	180	184	96	96
MEDIUM	35	6 Med	180	180	99	99
MEDIUM	1	7 Med	182	182	99	99
MEDIUM	2	7 Med	180	180	99	99
MEDIUM	3	7 Med	180	180	96	99
MEDIUM	4	7 Med	180	184	99	103
MEDIUM	5	7 Med	180	180	99	103
MEDIUM	6	7 Med	180	180	99	103

MEDIUM	7	7 Med	180	182	99	99
MEDIUM	8	7 Med	180	184	99	99
MEDIUM	9	7 Med	180	180	99	103
MEDIUM	10	7 Med	180	182	99	103
MEDIUM	11	7 Med	182	184	99	99
MEDIUM	12	7 Med	180	184	99	99
MEDIUM	13	7 Med	184	184	96	103
MEDIUM	14	7 Med	180	184	99	103
MEDIUM	15	7 Med	180	184	99	103
MEDIUM	16	7 Med	180	180	103	103
MEDIUM	17	7 Med	180	180	103	103
MEDIUM	18	7 Med	180	180	103	103
MEDIUM	19	7 Med	180	180	103	103
MEDIUM	20	7 Med	180	182	96	99
MEDIUM	21	7 Med	180	184	99	99
MEDIUM	22	7 Med	180	180	99	99
MEDIUM	23	7 Med	180	180	99	99
MEDIUM	24	7 Med	180	180	99	99
MEDIUM	25	7 Med	180	180	99	99
MEDIUM	26	7 Med	180	180	99	99
MEDIUM	27	7 Med	180	180	96	99
MEDIUM	28	7 Med	180	184	99	99
MEDIUM	29	7 Med	180	184	96	99
MEDIUM	1	8 Med	180	184	96	96
MEDIUM	2	8 Med	180	184	99	99
MEDIUM	3	8 Med	180	184	99	103
MEDIUM	4	8 Med	180	184	96	99
MEDIUM	5	8 Med	184	184	96	96
MEDIUM	6	8 Med	180	184	93	99
MEDIUM	7	8 Med	180	180	99	99
MEDIUM	8	8 Med	182	182	96	99
MEDIUM	9	8 Med	182	182	99	99
MEDIUM	10	8 Med	-9	-9	-9	-9
MEDIUM	11	8 Med	180	180	99	99
MEDIUM	12	8 Med	180	180	93	103
MEDIUM	13	8 Med	180	184	93	103
MEDIUM	14	8 Med	180	184	93	103
MEDIUM	15	8 Med	180	184	93	93
MEDIUM	16	8 Med	180	184	96	99
MEDIUM	17	8 Med	180	184	99	99
MEDIUM	18	8 Med	180	180	99	99
MEDIUM	19	8 Med	180	184	99	99
MEDIUM	20	8 Med	182	184	99	99
MEDIUM	21	8 Med	180	184	99	99
MEDIUM	22	8 Med	180	184	99	99
MEDIUM	23	8 Med	180	184	99	99
MEDIUM	24	8 Med	180	184	93	99
MEDIUM	25	8 Med	180	184	96	99
MEDIUM	26	8 Med	180	180	99	99

MEDIUM	27	8 Med	180	184	99	99
MEDIUM	28	8 Med	180	180	99	103
MEDIUM	29	8 Med	180	182	96	99
MEDIUM	30	8 Med	184	184	99	99
MEDIUM	31	8 Med	180	184	93	99
MEDIUM	32	8 Med	180	182	99	99
MEDIUM	33	8 Med	180	182	99	99
LARGE	1	9 Lrg	180	184	99	103
LARGE	2	9 Lrg	180	184	99	103
LARGE	3	9 Lrg	180	184	99	103
LARGE	4	9 Lrg	180	180	93	99
LARGE	5	9 Lrg	180	180	96	96
LARGE	6	9 Lrg	180	180	96	99
LARGE	7	9 Lrg	180	182	96	99
LARGE	8	9 Lrg	180	180	103	103
LARGE	9	9 Lrg	180	182	99	99
LARGE	10	9 Lrg	180	180	99	99
LARGE	11	9 Lrg	180	180	96	96
LARGE	12	9 Lrg	180	182	93	99
LARGE	13	9 Lrg	180	184	96	99
LARGE	14	9 Lrg	180	180	99	103
LARGE	15	9 Lrg	182	184	99	99
LARGE	16	9 Lrg	180	184	99	103
LARGE	17	9 Lrg	-9	-9	-9	-9
LARGE	18	9 Lrg	184	184	99	99
LARGE	19	9 Lrg	180	184	93	99
LARGE	20	9 Lrg	180	180	99	99
LARGE	21	9 Lrg	182	182	96	103
LARGE	22	9 Lrg	180	180	99	99
LARGE	23	9 Lrg	180	184	99	103
LARGE	24	9 Lrg	180	184	96	99
LARGE	25	9 Lrg	182	182	96	103
LARGE	26	9 Lrg	180	180	99	99
LARGE	27	9 Lrg	180	180	103	103
LARGE	28	9 Lrg	180	180	93	103
LARGE	29	9 Lrg	180	184	99	99
LARGE	30	9 Lrg	180	182	93	99
LARGE	31	9 Lrg	180	182	99	99
LARGE	32	9 Lrg	180	180	99	103
LARGE	33	9 Lrg	180	182	96	99
LARGE	34	9 Lrg	180	184	99	103
LARGE	35	9 Lrg	180	180	103	103
LARGE	36	9 Lrg	180	180	99	99
LARGE	37	9 Lrg	180	182	99	103
LARGE	38	9 Lrg	180	184	96	103
LARGE	39	9 Lrg	180	182	96	99
LARGE	1	10 Lrg	180	184	99	99
LARGE	2	10 Lrg	180	184	99	99
LARGE	3	10 Lrg	184	184	99	103

LARGE	4	10 Lrg	180	180	99	99
LARGE	5	10 Lrg	180	184	99	99
LARGE	6	10 Lrg	-9	-9	-9	-9
LARGE	7	10 Lrg	180	184	96	99
LARGE	8	10 Lrg	180	180	99	99
LARGE	9	10 Lrg	180	180	96	96
LARGE	10	10 Lrg	180	180	96	96
LARGE	11	10 Lrg	180	184	96	103
LARGE	12	10 Lrg	180	180	99	99
LARGE	13	10 Lrg	180	182	96	103
LARGE	14	10 Lrg	180	182	93	96
LARGE	15	10 Lrg	180	184	99	103
LARGE	16	10 Lrg	180	180	99	99
LARGE	17	10 Lrg	180	182	99	99
LARGE	18	10 Lrg	184	184	99	99
LARGE	19	10 Lrg	180	180	99	103
LARGE	20	10 Lrg	180	180	99	99
LARGE	21	10 Lrg	178	180	96	99
LARGE	22	10 Lrg	180	184	99	99
LARGE	23	10 Lrg	180	180	99	99
LARGE	24	10 Lrg	180	180	99	99
LARGE	25	10 Lrg	180	180	93	99
LARGE	26	10 Lrg	180	184	99	99
LARGE	27	10 Lrg	180	184	99	99
LARGE	28	10 Lrg	180	180	99	99
LARGE	29	10 Lrg	180	184	99	99
LARGE	30	10 Lrg	180	180	99	99
LARGE	31	10 Lrg	180	180	99	99
LARGE	32	10 Lrg	180	182	99	103
LARGE	33	10 Lrg	180	180	96	99
LARGE	34	10 Lrg	180	186	96	103
LARGE	35	10 Lrg	180	180	96	96
LARGE	36	10 Lrg	-9	-9	-9	-9
LARGE	37	10 Lrg	180	180	99	99
LARGE	38	10 Lrg	180	184	99	99
LARGE	1	11 Lrg	182	182	96	99
LARGE	2	11 Lrg	180	182	99	103
LARGE	3	11 Lrg	180	180	103	103
LARGE	4	11 Lrg	180	184	93	96
LARGE	5	11 Lrg	180	184	96	99
LARGE	6	11 Lrg	180	184	99	103
LARGE	7	11 Lrg	182	184	93	96
LARGE	8	11 Lrg	180	182	93	103
LARGE	9	11 Lrg	180	180	96	96
LARGE	10	11 Lrg	180	184	99	99
LARGE	11	11 Lrg	180	180	96	96
LARGE	12	11 Lrg	180	184	99	103
LARGE	13	11 Lrg	180	184	99	103
LARGE	14	11 Lrg	180	184	99	99

LARGE	15	11 Lrg	180	184	96	99
LARGE	16	11 Lrg	182	184	99	103
LARGE	17	11 Lrg	182	184	96	99
LARGE	18	11 Lrg	180	184	99	99
LARGE	19	11 Lrg	180	184	99	99
LARGE	20	11 Lrg	180	184	99	103
LARGE	21	11 Lrg	180	182	96	99
LARGE	22	11 Lrg	182	184	99	103
LARGE	23	11 Lrg	180	184	99	99
LARGE	24	11 Lrg	180	184	96	99
LARGE	25	11 Lrg	180	184	99	99
LARGE	26	11 Lrg	180	184	96	99
LARGE	27	11 Lrg	182	182	99	99
LARGE	28	11 Lrg	182	184	96	99
LARGE	29	11 Lrg	182	182	99	99
LARGE	30	11 Lrg	180	184	99	99
LARGE	31	11 Lrg	180	184	96	99
LARGE	32	11 Lrg	180	184	99	103
LARGE	1	12 Lrg	180	184	96	99
LARGE	2	12 Lrg	180	180	99	103
LARGE	3	12 Lrg	180	180	96	96
LARGE	4	12 Lrg	180	180	90	96
LARGE	5	12 Lrg	180	180	96	99
LARGE	6	12 Lrg	180	180	96	96
LARGE	7	12 Lrg	180	184	93	99
LARGE	8	12 Lrg	180	180	99	103
LARGE	9	12 Lrg	180	184	96	103
LARGE	10	12 Lrg	180	180	103	103
LARGE	11	12 Lrg	180	180	99	103
LARGE	12	12 Lrg	180	180	99	99
LARGE	13	12 Lrg	180	180	96	103
LARGE	14	12 Lrg	180	184	93	99
LARGE	15	12 Lrg	180	180	99	103
LARGE	16	12 Lrg	180	184	96	99
LARGE	17	12 Lrg	180	184	99	99
LARGE	18	12 Lrg	180	180	99	103
LARGE	19	12 Lrg	180	180	99	99
LARGE	20	12 Lrg	180	180	99	103
LARGE	21	12 Lrg	180	180	96	99
LARGE	22	12 Lrg	180	180	99	99
LARGE	23	12 Lrg	180	180	93	103
LARGE	24	12 Lrg	180	184	96	99
LARGE	25	12 Lrg	180	180	96	103
LARGE	26	12 Lrg	180	180	96	96
LARGE	27	12 Lrg	180	180	99	103
LARGE	28	12 Lrg	180	180	99	103
LARGE	29	12 Lrg	180	180	96	99

Primer 3		Primer 4		Primer 5		Primer 6		Primer 7
4343-1	4343-2	1233-1	1233-2	4914-1	4914-2	1810-1	1810-2	4483-1
160	163	98	102	114	116	180	183	186
152	160	100	105	118	120	180	183	182
152	156	96	100	114	116	177	180	188
163	163	98	98	114	120	177	180	188
152	163	98	105	118	120	180	183	182
160	163	96	105	114	114	180	183	186
152	152	102	102	114	114	180	183	186
160	160	96	96	114	114	180	183	188
160	160	96	102	120	120	180	183	186
160	160	100	107	114	120	180	183	188
163	163	96	96	118	120	177	180	186
160	160	96	98	114	116	180	183	188
160	163	96	96	114	116	180	183	186
160	163	100	102	114	116	180	183	182
160	163	100	100	114	120	180	183	186
152	163	100	105	114	120	180	183	182
163	163	102	105	114	114	177	180	182
163	163	98	98	114	114	180	183	182
160	160	105	105	120	120	180	183	186
152	163	96	102	118	120	180	180	182
152	163	98	100	114	120	180	183	188
152	152	102	102	114	114	180	183	186
152	158	96	100	114	116	180	183	188
152	152	96	100	114	120	177	180	182
152	152	96	96	114	120	180	183	188
160	163	98	98	116	118	180	183	188
152	152	100	100	116	118	177	180	182
152	160	96	102	114	116	180	183	182
152	160	100	102	116	120	180	183	188
160	163	100	107	114	114	180	183	182
152	160	102	107	114	120	180	183	186
160	163	105	107	116	116	177	180	182
152	160	100	102	114	114	180	183	182
152	163	102	105	114	120	180	180	182
163	172	100	100	116	118	180	183	186
154	154	98	100	114	118	177	180	186
152	160	98	98	114	120	180	183	182
152	160	98	100	114	114	180	183	182
152	152	98	100	114	118	177	180	194
160	163	102	105	114	114	180	183	182
163	163	100	118	114	118	180	183	182
160	160	96	98	114	116	180	183	180
160	160	100	100	114	114	177	186	180
152	152	100	100	116	120	180	183	186
152	163	100	107	114	114	180	183	204
163	163	100	100	114	118	180	182	186
152	160	100	100	114	118	180	183	200

152	160	98	100	114	114	183	183	200
152	160	98	100	114	114	180	183	188
163	172	98	100	114	118	180	180	188
163	163	98	100	114	116	180	183	180
163	163	98	100	114	116	180	183	180
152	160	100	100	114	114	180	183	180
152	163	98	98	114	114	180	183	188
152	172	100	102	114	114	180	183	186
160	163	98	100	114	116	180	183	190
160	163	100	100	114	116	180	183	190
152	152	100	105	114	118	180	183	186
152	160	98	100	114	118	177	180	182
152	160	98	102	114	114	180	183	186
163	160	100	100	114	114	180	183	190
152	152	100	102	114	114	180	183	188
152	160	98	100	114	114	194	200	182
152	152	100	100	114	114	180	183	188
152	163	98	102	114	118	180	183	186
154	160	100	100	114	116	180	183	174
160	160	98	98	114	114	180	180	174
160	160	100	100	114	116	180	183	188
154	160	100	100	116	116	180	183	174
154	160	100	100	114	116	180	183	174
154	154	98	98	116	116	180	186	174
160	160	100	100	114	116	177	177	174
160	160	100	100	114	116	180	183	174
154	154	100	100	114	116	180	183	174
160	160	100	102	114	116	180	183	190
160	160	100	100	116	116	177	180	174
152	154	100	102	114	114	180	183	188
152	152	96	98	114	114	177	183	188
-9	-9	-9	-9	-9	-9	177	183	188
163	163	100	102	114	114	177	180	182
160	160	100	100	118	118	180	183	174
163	163	98	100	114	114	177	183	186
163	163	100	102	114	114	177	180	182
160	160	100	100	114	118	180	183	180
160	160	96	100	114	114	177	183	188
163	163	98	100	114	114	183	188	182
160	163	100	100	114	114	180	183	174
160	160	100	100	114	114	180	183	188
152	152	96	96	114	114	183	188	174
154	154	100	102	114	114	180	183	182
160	160	98	102	118	118	180	183	174
152	166	100	100	114	114	180	180	180
160	160	98	98	114	118	180	183	188
160	160	98	98	114	118	180	183	188
163	163	96	98	114	116	177	183	186
163	163	100	102	114	114	-9	-9	-9

163	163	98	100	114	118	183	183	186
152	158	98	100	114	116	180	183	174
154	154	96	98	114	118	180	183	192
163	163	102	102	114	116	177	183	174
158	163	100	102	114	118	180	183	180
152	158	98	100	114	116	177	183	182
152	152	100	100	114	114	177	183	180
160	163	100	100	114	114	180	183	186
152	163	100	100	114	116	180	183	174
152	152	100	100	114	114	177	180	174
163	163	100	102	114	116	177	183	174
163	163	98	100	114	118	180	183	188
152	152	100	100	114	114	177	183	174
152	158	100	100	114	118	177	180	180
152	166	96	98	118	118	177	177	180
160	163	100	100	114	118	180	183	182
163	163	98	102	114	114	180	183	174
170	170	98	100	114	118	177	180	180
152	152	100	105	114	118	177	183	174
154	163	100	102	114	114	180	183	182
160	166	98	100	114	114	180	183	188
163	166	100	100	114	116	180	180	182
160	166	98	100	114	114	180	183	188
152	163	98	98	114	114	177	183	188
152	163	98	100	114	114	180	183	188
163	163	98	102	114	116	180	183	182
160	160	98	98	118	120	180	183	182
152	160	98	105	114	114	180	183	182
163	163	100	102	114	122	180	183	188
154	158	98	102	114	118	177	180	188
154	158	98	102	118	118	177	183	188
160	169	98	100	114	116	180	183	186
160	169	98	100	114	114	180	183	188
169	169	98	102	114	116	177	180	178
152	158	98	105	114	118	177	183	188
160	163	98	100	114	118	177	183	182
160	160	96	98	116	118	177	183	182
152	160	98	100	116	120	180	183	188
152	160	96	98	116	120	180	183	188
152	154	100	100	114	118	180	183	188
152	154	98	100	118	118	177	180	188
152	163	98	98	114	116	180	183	182
152	158	98	100	114	118	180	183	188
160	163	96	100	116	118	180	183	182
152	152	100	100	120	120	180	183	180
160	169	98	98	114	118	180	183	188
152	152	98	98	116	118	180	183	182
152	160	98	100	114	118	177	186	182
154	158	98	100	114	118	180	186	182

152	163	102	105	120	120	180	183	188
152	169	100	102	118	118	180	183	182
154	160	98	100	114	114	177	183	182
160	160	98	98	118	120	180	183	182
154	158	98	102	120	120	177	183	192
152	152	100	102	114	118	180	183	182
152	152	100	102	114	118	180	183	182
152	163	98	98	114	116	180	183	182
163	163	100	100	114	114	177	177	188
160	160	96	98	114	114	177	183	188
160	163	100	100	114	114	180	180	190
152	152	105	105	114	114	177	180	188
154	163	100	100	118	118	180	183	188
163	163	100	102	116	120	180	183	182
170	170	118	123	131	131	177	180	180
158	158	102	105	114	114	177	177	182
152	163	102	105	114	116	177	180	182
152	152	100	100	114	114	183	186	190
152	160	98	100	114	116	183	183	188
152	152	100	100	116	116	177	180	188
163	163	100	107	114	118	177	180	188
160	163	100	100	116	120	177	183	185
163	163	100	102	114	116	177	180	188
163	163	100	102	99	114	177	183	188
160	160	102	102	116	118	177	183	188
158	158	102	105	114	116	177	183	182
152	152	98	98	114	116	177	183	188
152	152	98	98	114	116	177	183	188
152	152	100	105	114	116	177	183	188
160	163	98	100	114	114	177	183	182
160	160	100	102	114	114	180	183	182
152	163	98	100	114	116	180	183	188
152	160	98	100	114	116	180	180	182
152	163	98	102	114	116	177	183	188
154	160	105	105	118	120	180	180	188
160	163	100	100	114	122	177	183	188
155	169	100	100	114	114	177	183	190
160	160	96	98	114	114	177	183	188
152	160	100	104	114	116	180	183	188
160	163	100	102	114	114	180	183	188
160	162	98	100	114	118	180	183	188
150	150	100	102	114	114	180	183	188
160	160	100	100	114	114	-9	-9	-9
152	160	100	100	116	120	180	183	188
152	163	102	102	116	118	177	183	188
160	160	100	102	118	118	180	183	190
160	160	98	98	114	114	177	180	182
152	160	96	98	114	114	177	180	190
152	163	98	102	114	114	180	183	203

152	160	100	102	120	120	180	183	182
152	160	98	100	114	114	180	183	188
152	160	98	105	114	116	180	183	188
160	163	100	102	114	116	180	183	188
160	160	102	102	114	122	180	183	188
152	160	100	100	114	114	180	183	188
160	160	100	100	114	114	177	180	188
160	160	100	105	114	116	177	180	188
160	160	100	102	114	114	180	183	188
160	163	105	105	114	116	180	183	180
152	152	100	100	116	116	180	183	192
152	160	100	107	114	116	180	183	188
152	163	100	100	114	118	180	183	182
-9	-9	100	107	114	114	180	183	182
152	152	98	102	114	114	180	183	188
152	163	98	100	114	114	180	183	188
160	160	98	100	114	118	180	183	188
152	160	98	100	114	114	180	183	188
152	160	98	100	114	114	-9	-9	182
152	152	98	98	114	114	180	183	188
152	160	100	100	114	114	177	180	188
-9	-9	-9	-9	114	114	-9	-9	-9
152	160	98	102	114	114	180	183	188
160	163	100	102	114	116	180	183	188
152	163	96	96	114	118	180	183	188
160	163	98	100	114	118	177	180	182
152	163	100	102	114	114	180	183	182
152	152	100	102	114	114	180	183	182
163	163	98	100	114	118	180	183	182
163	172	100	100	116	120	180	183	182
163	172	100	100	116	120	180	183	182
160	163	100	102	118	118	177	183	182
-9	-9	-9	-9	-9	-9	180	183	182
163	163	96	96	118	122	180	183	186
160	163	98	100	114	120	180	183	186
160	163	98	100	114	120	180	183	188
160	163	98	100	114	120	180	183	188
160	160	98	100	114	114	180	183	188
163	163	100	102	114	114	180	183	182
152	163	100	102	114	114	180	183	182
152	163	98	100	114	118	180	183	188
163	163	100	102	114	114	180	183	186
163	163	102	102	114	118	177	180	182
152	152	100	100	114	116	180	183	182
152	163	100	102	114	116	177	180	182
152	160	98	102	114	118	177	180	182
152	152	96	102	114	118	177	180	182
154	163	98	100	114	120	177	180	192
-9	-9	-9	-9	-9	-9	180	183	186

152	154	96	102	114	118	180	183	188
152	163	98	102	114	118	180	183	-9
152	163	98	102	114	114	180	183	186
152	163	96	100	114	116	177	180	192
163	166	100	102	114	116	180	183	186
152	163	96	98	114	118	177	180	188
152	163	100	106	120	120	180	183	186
152	163	98	98	118	120	177	180	190
160	163	100	100	118	118	177	180	188
158	163	98	98	116	118	180	183	182
160	163	100	100	118	118	180	183	188
160	163	96	98	114	116	177	180	188
154	160	98	98	114	116	180	183	188
160	163	98	100	114	114	180	183	190
152	152	100	105	114	116	177	180	182
152	163	100	100	114	118	180	183	188
152	163	98	100	114	114	180	180	188
152	152	100	102	114	114	183	186	188
152	166	100	100	114	116	177	183	182
152	163	102	105	114	120	180	183	182
152	163	100	100	114	118	180	183	188
160	163	100	105	118	118	180	183	188
160	163	98	100	114	116	183	186	188
-9	-9	-9	-9	-9	-9	-9	-9	-9
152	152	96	96	114	114	180	183	190
150	150	100	102	-9	-9	-9	-9	-9
163	163	100	102	118	118	180	183	188
160	160	100	102	114	118	180	183	188
152	163	98	98	114	118	-9	-9	-9
160	163	100	100	114	118	180	183	188
152	152	100	102	114	118	180	186	186
152	163	100	100	118	118	180	183	188
152	170	96	100	114	114	177	180	188
152	160	100	100	114	118	180	183	184
152	160	98	100	114	114	180	183	186
154	160	100	102	114	114	177	180	186
152	163	100	105	114	118	180	186	178
152	160	100	102	114	114	180	183	178
152	152	98	102	114	114	-9	-9	178
160	160	98	100	118	118	180	183	182
152	160	100	105	114	114	183	186	182
152	160	100	100	114	118	177	180	188
160	160	98	98	114	114	177	183	182
160	163	98	100	118	120	180	191	194
150	163	105	105	114	114	177	177	186
153	160	98	102	114	118	177	183	182
-9	-9	-9	-9	114	114	177	180	186
160	163	98	100	114	116	180	183	180
152	163	98	100	114	116	180	183	190

152	160	98	98	114	114	177	183	182
163	163	100	100	114	118	180	183	182
-9	-9	-9	-9	-9	-9	180	183	188
152	163	98	102	114	120	177	180	182
163	163	102	100	114	114	177	183	186
163	163	102	105	114	114	180	183	182
163	163	102	105	114	114	180	183	182
152	163	100	100	114	118	180	183	188
163	163	98	100	114	118	180	183	188
163	170	100	100	114	118	180	180	180
152	163	98	105	114	120	177	183	188
163	163	98	100	118	118	180	183	188
163	163	102	105	114	114	183	186	188
154	166	98	102	114	118	177	180	186
154	158	100	102	114	114	183	186	182
152	170	100	100	114	114	180	183	182
163	163	98	98	114	118	180	183	186
163	163	98	102	114	114	180	183	180
154	163	100	100	114	118	177	180	186
152	152	98	100	114	118	177	180	182
163	163	98	98	114	114	180	183	188
152	170	105	107	114	116	180	183	188
152	163	98	105	114	118	177	180	182
152	163	100	100	114	118	180	183	182
152	163	100	102	118	118	180	183	182
163	163	100	100	114	116	180	180	182
163	163	100	100	114	114	180	180	188
152	152	100	105	118	118	180	183	182
152	163	98	105	114	114	180	183	188
158	163	98	100	118	120	180	183	182
150	160	100	98	114	114	177	180	182
163	163	100	105	114	114	180	183	182
-9	-9	-9	-9	-9	-9	-9	-9	-9
156	156	98	98	114	120	-9	-9	-9
163	163	100	100	114	118	-9	-9	182
152	163	98	100	114	118	180	183	188
152	160	100	102	114	120	180	183	182
152	152	102	105	114	114	183	183	194
152	163	100	100	114	118	177	180	182
152	160	100	100	114	114	177	180	186
160	163	102	105	114	118	177	180	182
152	163	100	105	114	114	177	180	182
152	163	98	105	114	122	177	183	188
163	163	98	100	114	114	180	183	182
152	152	98	100	114	116	180	183	188
160	163	100	105	114	114	180	183	188
152	152	98	100	114	114	180	183	188
152	160	100	100	114	114	177	180	186
152	152	98	100	114	114	180	183	186

152	163	98	100	114	118	180	183	190
160	160	98	98	114	118	177	177	182
152	152	100	100	114	116	177	180	186
152	152	98	100	114	116	180	180	186
160	163	98	100	114	114	180	183	188
160	163	98	100	114	118	183	183	188
152	152	98	100	114	122	177	183	188
152	154	98	100	114	116	180	183	182
152	160	98	100	114	114	177	183	182
152	163	100	100	114	114	180	183	182
152	163	98	100	114	114	180	183	188
154	158	98	100	114	116	180	183	186
160	163	98	98	114	116	180	183	188
154	158	98	102	114	114	180	183	188
160	163	98	98	114	116	180	183	188
163	163	98	102	114	116	180	183	182
160	163	98	100	114	116	180	183	182
152	160	100	102	114	120	180	183	182
152	152	100	100	114	114	180	183	188
160	163	98	100	114	116	180	183	182
160	163	100	100	114	114	180	183	188
160	163	100	100	114	114	180	183	188
152	152	100	102	114	118	177	180	188
152	152	100	100	114	118	177	180	182
163	163	98	100	114	114	180	183	186
152	163	98	100	114	118	180	183	182
160	163	100	105	114	114	180	183	188
152	152	98	100	118	118	177	183	188
152	158	98	100	114	114	180	183	180
158	158	102	102	114	118	177	183	188
163	163	100	100	116	116	180	183	186
152	163	98	100	116	116	180	183	186
163	163	107	107	120	120	180	182	186
152	163	98	100	114	116	180	182	180
152	152	98	98	114	114	180	183	188
152	163	98	100	114	116	177	183	188
158	163	100	102	114	114	177	180	188
163	163	98	100	114	118	180	183	182
163	163	98	100	114	116	180	183	180
160	172	98	100	114	118	180	183	180
160	163	100	100	114	118	177	180	190
152	163	98	102	114	114	177	183	186
152	152	100	100	116	118	180	183	182
163	163	100	100	114	118	180	183	188
152	152	100	102	114	114	180	183	182
152	152	100	100	114	114	180	183	182
152	163	100	100	114	116	180	183	188

	Primer 8		Primer 9		Primer 10		Primer 11	
4483-2	5250-1	5250-2	5441-1	5441-2	1808-1	1808-2	4536-1	4536-2
188	190	190	217	219	294	300	193	193
188	186	205	213	215	294	300	193	193
190	186	186	221	-9	-9	-9	187	187
188	186	194	213	221	300	303	193	193
188	184	190	213	213	300	300	193	193
188	184	186	-9	-9	300	300	193	193
188	186	186	-9	-9	-9	-9	-9	-9
188	186	190	213	215	300	300	193	193
188	186	190	209	209	300	303	193	193
188	186	204	217	217	300	300	176	215
188	190	190	213	213	300	300	193	193
188	106	204	215	215	300	300	193	193
188	190	190	215	215	294	300	185	193
182	186	186	209	211	300	300	193	193
190	186	186	215	215	300	312	193	193
188	186	186	-9	-9	-9	-9	193	193
188	184	186	213	215	300	300	193	193
182	194	196	-9	-9	-9	-9	-9	-9
188	184	204	-9	-9	-9	-9	-9	-9
188	184	190	213	213	300	300	193	193
188	186	186	219	221	300	309	193	193
186	184	204	213	215	297	300	187	193
188	184	184	219	235	294	294	193	193
182	194	196	211	215	297	300	153	193
188	184	184	215	215	294	300	187	193
188	184	194	-9	-9	-9	-9	-9	-9
186	186	188	-9	-9	-9	-9	199	199
186	184	190	209	211	300	300	187	193
188	184	190	217	219	309	312	193	193
188	184	186	205	205	300	309	176	218
188	184	204	211	215	294	309	193	193
182	190	190	211	211	300	300	193	193
188	186	186	215	215	300	303	185	193
188	0	0	215	215	300	300	187	193
188	184	188	205	213	297	297	193	193
186	188	190	213	215	300	300	193	193
188	184	194	215	215	300	303	193	193
188	186	186	213	215	300	300	193	193
204	198	198	-9	-9	-9	-9	-9	-9
186	184	186	213	215	300	300	193	193
186	184	184	211	213	300	300	185	193
186	184	186	213	217	300	300	193	193
186	184	184	219	219	294	294	193	193
188	184	186	215	215	300	309	193	193
204	188	190	215	215	294	300	193	193
188	184	184	205	205	300	300	187	193
204	204	207	205	211	300	300	187	193

204	207	207	217	219	294	294	193	193
188	184	184	213	215	300	300	193	193
188	184	186	213	215	294	294	193	193
188	186	204	215	217	300	303	193	193
188	186	204	215	217	300	303	193	193
188	186	186	-9	-9	294	300	187	187
188	190	204	211	213	300	309	193	193
188	186	186	211	213	300	312	193	193
190	184	186	213	215	300	309	193	193
190	184	186	205	215	300	303	193	193
190	186	186	205	215	300	303	193	193
190	186	186	205	207	300	300	187	193
190	186	186	205	207	300	300	193	193
190	184	186	-9	-9	-9	-9	-9	-9
188	186	186	-9	-9	-9	-9	-9	-9
188	188	188	211	213	297	297	193	193
188	207	207	213	215	300	300	187	193
188	184	184	211	213	294	300	193	199
188	184	184	-9	-9	-9	-9	-9	-9
188	184	184	213	213	300	306	199	199
188	184	184	-9	-9	-9	-9	199	199
188	184	184	205	205	-9	-9	199	199
188	184	184	213	215	-9	-9	187	193
188	184	184	205	205	297	300	187	193
188	184	184	205	205	297	297	193	193
188	-9	-9	205	205	297	297	187	193
188	184	184	215	215	-9	-9	193	193
190	-9	-9	-9	-9	-9	-9	130	130
188	192	192	211	211	300	300	193	199
188	184	184	205	205	-9	-9	193	193
192	-9	-9	-9	-9	-9	-9	193	193
194	-9	-9	205	207	300	300	183	199
188	184	186	205	207	300	300	183	193
182	184	184	215	217	294	306	183	183
186	184	186	203	203	300	300	183	193
188	184	186	209	209	300	300	183	193
188	186	186	205	213	-9	-9	193	193
188	186	186	-9	-9	294	294	193	193
182	184	184	215	215	300	300	183	193
182	184	184	213	215	294	300	183	193
188	184	184	213	215	294	294	193	193
182	184	184	213	215	300	300	183	193
188	184	184	-9	-9	-9	-9	183	193
182	184	184	207	207	297	300	193	193
188	184	184	215	225	300	300	183	193
190	184	188	215	225	300	300	193	193
190	184	188	215	225	315	325	193	193
180	184	184	223	225	300	300	187	187
-9	184	184	215	215	300	300	193	193

188	207	207	213	215	303	303	193	193
192	184	190	213	225	294	300	193	193
192	184	184	213	225	-9	-9	193	193
180	184	184	213	213	300	300	193	193
192	184	186	211	217	294	300	193	193
188	184	184	205	205	300	303	183	193
192	184	186	209	211	300	303	193	193
188	184	184	211	213	291	300	187	193
182	184	186	215	217	300	300	187	193
180	184	184	213	215	-9	-9	187	193
180	184	184	213	215	-9	-9	193	193
190	184	184	209	215	291	300	187	193
180	184	184	223	225	300	303	187	193
190	184	184	213	225	300	303	187	193
188	184	184	213	217	300	300	193	193
188	184	184	203	207	300	300	193	193
180	184	184	211	211	297	300	187	193
182	184	186	201	205	300	303	193	193
182	184	194	205	207	297	303	187	193
194	184	194	207	209	297	297	187	199
192	184	184	205	205	300	303	-9	-9
188	184	184	205	207	297	300	187	193
192	184	184	205	205	300	303	187	193
190	184	186	207	215	300	300	187	193
188	184	186	-9	-9	-9	-9	-9	-9
188	184	184	-9	-9	-9	-9	-9	-9
188	184	184	215	217	300	300	193	193
188	184	184	223	225	294	300	187	193
188	186	194	213	215	294	294	193	193
192	186	194	217	229	300	300	193	199
192	194	194	215	217	298	300	193	199
188	186	186	213	215	300	303	187	193
188	186	186	213	215	300	300	193	193
178	190	190	213	215	300	309	193	193
188	186	186	215	217	-9	-9	193	193
190	186	190	213	217	303	309	193	193
190	186	190	209	217	309	309	193	193
192	184	184	-9	-9	-9	-9	-9	-9
192	184	184	213	215	300	300	193	193
188	186	186	215	217	294	294	193	193
188	186	186	215	217	294	294	193	193
188	186	186	209	213	300	300	193	193
192	186	188	213	213	300	300	193	193
186	186	186	211	213	300	303	187	193
190	186	188	213	215	294	300	193	193
188	186	186	213	215	-9	-9	193	193
182	186	186	213	215	300	300	187	193
188	186	190	211	213	292	300	187	193
188	186	186	211	217	300	303	187	193

188	186	190	213	217	300	300	187	193
182	186	186	209	213	300	300	193	193
192	184	186	209	217	300	300	193	193
188	186	186	215	217	300	300	193	193
192	194	194	211	213	300	300	187	193
186	184	186	209	213	300	309	193	193
182	184	184	209	213	300	309	193	193
188	184	188	211	211	294	309	193	193
188	184	186	215	217	-9	-9	193	193
188	194	194	209	233	309	309	193	193
190	188	188	211	213	300	300	193	193
188	186	186	207	209	300	300	187	187
188	184	190	217	219	300	309	187	193
186	184	184	209	213	300	300	193	193
188	186	186	215	233	297	300	187	193
182	-9	-9	213	213	300	300	187	193
192	186	186	-9	-9	-9	-9	193	193
190	186	186	213	213	300	300	193	193
192	188	192	209	209	294	300	193	193
188	186	186	209	209	300	300	193	193
188	184	188	209	209	300	300	187	193
185	186	186	209	209	300	300	193	193
188	184	184	209	213	300	300	187	193
188	194	194	213	215	300	300	187	193
192	186	190	209	215	294	300	193	193
182	-9	-9	211	213	300	300	187	193
190	186	186	211	213	300	309	187	193
190	186	186	215	217	306	309	193	193
190	186	186	-9	-9	-9	-9	-9	-9
190	184	184	209	213	300	309	187	193
182	186	186	217	217	297	300	-9	-9
188	180	184	217	217	300	300	184	193
188	184	186	229	231	300	300	187	193
190	184	186	213	215	300	312	187	193
188	184	188	213	215	300	300	184	193
190	188	188	213	215	294	309	193	193
190	192	192	213	215	294	309	193	193
188	194	194	209	233	294	300	193	193
190	186	192	213	213	300	303	193	193
192	188	190	203	203	300	303	193	193
188	186	190	213	224	300	300	193	193
188	186	186	215	217	300	300	184	193
-9	-9	-9	217	217	300	300	193	193
188	184	184	217	217	300	303	193	193
190	-9	-9	215	219	300	300	193	193
190	184	184	215	217	297	312	193	193
188	184	186	207	209	297	300	193	193
190	184	186	207	207	300	300	187	193
208	204	225	201	211	300	300	193	193

190	184	186	211	213	300	300	193	193
190	184	186	215	215	300	300	184	193
188	188	188	213	215	300	300	184	193
190	188	190	203	217	300	300	193	193
188	190	190	197	207	300	300	193	193
190	184	184	-9	-9	-9	-9	-9	-9
190	184	186	213	215	300	300	193	193
192	184	184	199	205	300	300	187	193
190	184	184	209	217	300	303	193	193
188	184	184	201	205	300	300	193	193
192	184	184	205	205	297	297	193	193
190	184	186	213	215	300	303	184	187
192	184	184	213	215	300	300	193	193
194	184	186	213	225	300	300	193	193
190	184	184	207	211	297	303	193	193
188	184	200	213	213	-9	-9	193	193
190	184	184	210	215	300	300	193	193
188	184	184	213	215	300	300	193	193
182	208	208	213	215	-9	-9	193	193
188	186	186	203	213	300	300	193	193
192	186	186	203	205	300	300	187	193
-9	-9	-9	213	215	-9	-9	193	193
190	184	190	-9	-9	-9	-9	-9	-9
192	186	190	209	209	300	300	193	193
188	194	194	209	217	294	300	187	193
188	186	188	213	217	300	303	193	193
188	188	188	205	205	300	303	193	193
188	188	188	273	275	300	306	193	193
190	194	194	273	275	300	306	193	193
188	184	186	207	209	297	300	193	193
188	184	186	207	209	297	300	193	193
182	184	186	211	225	297	300	187	193
182	184	186	109	109	300	300	193	193
186	186	186	211	217	297	300	193	193
188	186	194	205	207	300	303	193	193
188	186	194	209	213	300	306	193	193
188	186	194	203	205	300	303	193	193
188	186	186	209	209	300	300	193	193
188	184	186	211	219	300	300	193	193
182	186	186	246	248	300	303	193	193
188	186	186	209	211	303	309	153	193
192	184	190	211	219	300	303	187	193
188	184	186	203	207	300	306	193	193
188	188	190	211	211	297	309	193	193
188	184	186	203	205	303	306	193	193
190	184	194	203	203	297	303	193	193
188	186	186	203	209	300	303	187	193
194	184	186	203	207	297	303	187	193
188	186	186	203	205	300	300	193	193

188	186	186	213	227	300	309	187	193
-9	-9	-9	199	205	300	300	193	193
188	186	188	203	203	300	303	193	193
192	186	188	205	205	300	300	187	193
188	186	190	203	203	300	300	193	193
188	186	186	209	211	297	300	187	193
188	190	190	209	215	294	300	193	193
190	184	184	213	215	300	300	193	193
192	184	186	213	215	303	309	193	193
188	186	192	213	215	300	300	193	193
188	186	186	211	213	300	300	187	193
188	186	188	209	215	294	303	193	193
190	188	194	213	215	294	300	193	193
190	184	188	215	233	300	300	187	193
190	184	190	211	213	300	300	193	193
188	186	194	215	215	300	300	187	193
192	186	190	211	213	300	300	193	193
188	184	186	219	221	300	300	193	193
188	184	186	209	215	300	300	193	193
188	188	190	-9	-9	-9	-9	193	193
188	-9	-9	209	209	294	300	193	193
194	184	194	211	213	291	300	187	193
188	186	186	215	217	300	300	193	193
-9	-9	-9	213	215	300	300	187	193
190	186	186	223	225	300	300	193	193
-9	-9	-9	211	211	300	306	193	193
188	186	186	207	213	300	303	193	193
188	186	186	207	213	306	309	193	193
-9	-9	-9	211	213	297	300	187	193
188	186	186	213	213	300	300	193	193
190	186	190	213	219	297	300	193	193
188	186	190	213	215	318	318	193	193
190	186	192	215	233	300	303	193	193
198	186	186	213	225	285	303	193	193
188	188	188	215	217	300	303	187	193
188	184	184	211	215	300	309	193	193
182	190	196	215	217	294	300	187	193
182	186	186	213	215	297	306	193	193
182	188	190	215	219	303	303	193	193
186	184	188	209	211	300	300	193	193
192	188	190	207	215	303	303	187	193
192	184	190	211	211	294	294	187	193
186	186	190	215	217	300	300	193	193
198	184	190	211	211	300	300	193	193
188	186	186	213	219	294	300	193	193
190	188	190	209	213	300	300	193	193
188	186	186	203	207	-9	-9	193	193
194	186	186	209	213	300	300	187	193
194	184	188	205	207	297	300	187	193

192	194	194	205	205	297	300	187	187
188	186	186	207	207	297	300	193	193
188	188	188	213	213	300	300	193	193
188	186	186	203	205	-9	-9	193	193
188	182	186	211	215	297	300	193	193
188	186	190	205	213	300	300	193	193
188	186	190	203	209	300	300	193	193
188	186	186	213	217	303	312	215	215
188	186	186	215	231	294	294	193	193
188	184	188	203	205	300	305	187	193
188	182	186	205	205	297	300	193	193
188	186	186	213	215	297	300	193	193
188	188	188	207	213	294	294	187	193
188	186	186	213	219	297	300	193	193
182	186	186	203	205	300	300	193	193
188	182	186	213	215	300	306	193	193
188	182	186	205	205	300	300	187	193
192	186	186	207	207	300	300	187	193
188	188	192	205	217	300	300	187	190
188	186	186	205	209	-9	-9	190	193
190	186	186	205	205	297	300	187	193
188	186	186	215	217	300	300	193	193
188	186	194	213	215	300	309	193	193
188	186	186	205	205	297	300	187	193
182	186	186	213	213	309	309	184	190
192	184	186	219	241	309	312	193	193
190	184	186	207	207	297	300	193	193
192	186	186	205	209	-9	-9	190	190
190	186	186	-9	-9	300	305	187	193
190	186	186	205	207	300	300	187	193
188	186	186	-9	-9	-9	-9	-9	-9
188	186	194	-9	-9	-9	-9	-9	-9
-9	-9	-9	203	205	-9	-9	193	193
-9	-9	-9	-9	-9	-9	-9	-9	-9
188	186	186	207	209	297	300	193	193
192	184	190	213	215	300	300	187	193
194	184	186	215	217	300	300	187	193
194	184	184	209	209	294	300	187	193
194	184	188	215	215	300	300	193	193
188	188	188	213	213	294	297	193	193
188	186	186	221	221	300	300	193	193
188	184	186	213	215	300	303	187	193
190	188	188	213	215	300	303	193	193
190	184	188	215	217	300	300	184	196
192	184	194	-9	-9	300	300	193	193
192	184	188	213	215	300	300	187	193
192	186	186	215	215	300	300	193	193
188	184	186	213	213	297	300	193	193
192	184	186	213	213	297	297	193	193

194	184	184	213	215	294	300	193	193
188	184	186	233	235	300	300	193	193
188	186	186	209	209	294	294	193	193
188	186	186	215	221	300	300	193	193
188	188	190	-9	-9	-9	-9	193	193
190	184	186	-9	-9	294	300	193	193
190	184	186	213	213	294	300	193	193
188	184	186	215	217	300	307	184	193
188	186	186	213	213	300	300	193	193
188	186	188	215	217	294	300	193	193
188	184	184	213	215	294	300	187	193
188	184	192	-9	-9	300	300	193	193
192	184	192	213	221	300	300	193	193
188	184	186	-9	-9	-9	-9	193	193
192	186	192	213	221	300	300	193	193
188	186	190	237	237	294	300	193	193
188	184	186	-9	-9	300	300	193	193
192	184	186	215	217	300	300	193	193
188	192	192	213	215	-9	-9	-9	-9
188	184	207	213	213	294	300	193	193
194	184	184	213	213	294	300	193	193
194	184	184	213	213	294	300	193	193
188	184	190	213	213	294	300	193	193
188	186	188	-9	-9	-9	-9	193	193
188	184	192	213	213	300	300	193	193
188	207	207	213	219	297	300	193	193
188	186	186	213	229	300	300	193	193
190	186	186	213	221	300	300	187	193
182	186	186	213	215	300	300	193	193
188	186	186	-9	-9	-9	-9	193	193
188	188	194	211	239	294	303	187	193
188	-9	-9	211	213	300	300	187	193
188	-9	-9	213	213	305	305	187	193
188	186	186	213	213	300	303	193	193
188	186	186	215	215	300	300	193	193
194	194	196	213	213	303	303	193	193
188	186	186	213	213	-9	-9	193	193
188	184	188	213	217	294	300	187	193
180	190	190	215	219	294	294	193	193
190	186	190	213	215	300	300	193	193
194	186	194	213	213	300	303	193	193
188	-9	-9	-9	-9	-9	-9	193	193
188	184	186	215	221	300	303	193	193
188	194	194	213	215	300	300	193	193
188	-9	-9	209	219	300	309	193	193
194	186	207	209	219	300	309	193	193
188	188	194	213	213	300	300	193	193
