# Supplementary material for: Life history and past demography maintain genetic structure, outcrossing rate, contemporary pollen gene flow of an understory herb in a highly fragmented rainforest
Source: PeerJ. 2016 Dec 22;4:e2764. doi: 10.7717/peerj.2764 (PMC5183091; doi:10.7717/peerj.2764)
Supplement: Data S2 [file peerj-04-2764-s012.pdf]

			Primer 1		Primer 2		Primer 3
**Fragment size**	**Population**	Family	432-1	432-2	5409-1	5409-2	1233-1
LARGE	11 Lrg	ZCT12	116	124	184	184	120
LARGE	11 Lrg	ZCT12	120	124	184	184	118
LARGE	11 Lrg	ZCT12!	120	124	184	184	122
LARGE	11 Lrg	ZCT14	120	120	180	180	122
LARGE	11 Lrg	ZCT14	120	124	180	180	122
LARGE	11 Lrg	ZCT14	120	120	180	184	124
LARGE	11 Lrg	ZCT14	120	120	180	180	122
LARGE	11 Lrg	ZCT14!	120	124	180	180	-9
LARGE	11 Lrg	ZCT21	114	120	184	184	120
LARGE	11 Lrg	ZCT21	114	120	184	184	118
LARGE	11 Lrg	ZCT21	114	120	184	186	118
LARGE	11 Lrg	ZCT21	114	120	186	188	120
LARGE	11 Lrg	ZCT21	114	120	184	186	118
LARGE	11 Lrg	ZCT21!	114	120	184	186	120
LARGE	11 Lrg	ZCT25	116	120	184	186	120
LARGE	11 Lrg	ZCT25	116	120	184	186	120
LARGE	11 Lrg	ZCT25	116	120	184	184	120
LARGE	11 Lrg	ZCT25	120	122	184	184	120
LARGE	11 Lrg	ZCT25	116	120	184	186	122
LARGE	11 Lrg	ZCT25	114	120	184	186	122
LARGE	11 Lrg	ZCT25!	116	120	184	186	122
LARGE	11 Lrg	ZCT3	116	124	184	184	122
LARGE	11 Lrg	ZCT3!	120	124	184	184	124
LARGE	11 Lrg	ZCT39	116	120	184	184	118
LARGE	11 Lrg	ZCT39	116	120	184	184	126
LARGE	11 Lrg	ZCT39	116	120	184	184	124
LARGE	11 Lrg	ZCT39	116	120	184	184	124
LARGE	11 Lrg	ZCT39	116	120	184	184	124
LARGE	11 Lrg	ZCT39	116	120	184	184	122
LARGE	11 Lrg	ZCT39	116	116	184	184	118
LARGE	11 Lrg	ZCT39	114	120	184	186	124
LARGE	11 Lrg	ZCT39!	116	120	184	184	124
LARGE	9 Lrg	SII14A!	116	124	184	184	120
LARGE	9 Lrg	SII14A	-9	-9	184	184	120
LARGE	9 Lrg	SII14A	116	120	184	184	120
LARGE	9 Lrg	SII14A	112	116	184	184	120
LARGE	9 Lrg	SII14A	116	120	184	188	118
LARGE	9 Lrg	SII14A	116	124	184	186	120
LARGE	9 Lrg	SII14A	116	120	184	186	118
LARGE	9 Lrg	SII14A	116	120	184	186	118
LARGE	9 Lrg	SII14A	116	120	184	184	124
LARGE	9 Lrg	SII14A	116	120	184	184	124
LARGE	9 Lrg	SII14A	116	120	184	184	118
LARGE	9 Lrg	SII18	116	120	184	186	120
LARGE	9 Lrg	SII18	120	124	186	186	120
LARGE	9 Lrg	SII18	120	124	184	186	120
LARGE	9 Lrg	SII18	114	120	184	184	118

LARGE	9 Lrg	SII18	120	124	184	186	120
LARGE	9 Lrg	SII18	120	124	184	184	120
LARGE	9 Lrg	SII18!	120	124	184	186	120
LARGE	9 Lrg	SII19B	114	120	184	184	122
LARGE	9 Lrg	SII19B	116	120	184	184	122
LARGE	9 Lrg	SII19B	120	120	184	184	122
LARGE	9 Lrg	SII19B!	114	120	184	184	122
LARGE	9 Lrg	SII19B	114	120	184	188	118
LARGE	9 Lrg	SII20B!	114	120	184	184	122
LARGE	9 Lrg	SII20B	114	120	184	184	118
LARGE	9 Lrg	SII20B	114	120	184	188	118
LARGE	9 Lrg	SII20B	114	120	184	188	118
LARGE	9 Lrg	SII20B	114	120	184	188	122
LARGE	9 Lrg	SII20B	114	120	184	184	116
LARGE	9 Lrg	SII26	116	120	184	184	120
LARGE	9 Lrg	SII26	120	124	184	184	122
LARGE	9 Lrg	SII26	116	120	184	184	120
LARGE	9 Lrg	SII26	120	124	184	184	120
LARGE	9 Lrg	SII26	120	124	184	186	122
LARGE	9 Lrg	SII26	116	120	184	184	120
LARGE	9 Lrg	SII26	120	124	184	184	120
LARGE	9 Lrg	SII26	120	124	184	186	118
LARGE	9 Lrg	SII26!	120	124	184	184	118
LARGE	9 Lrg	SII35A	120	120	184	186	118
LARGE	9 Lrg	SII35A	120	120	184	186	118
LARGE	9 Lrg	SII35A	116	120	184	186	118
LARGE	9 Lrg	SII35A	120	120	184	186	118
LARGE	9 Lrg	SII35A	120	120	184	186	118
LARGE	9 Lrg	SII35A	120	120	184	188	116
LARGE	9 Lrg	SII35A!	120	120	184	186	118
LARGE	9 Lrg	SII4	120	120	188	188	122
LARGE	9 Lrg	SII4	120	120	184	188	122
LARGE	9 Lrg	SII4	116	120	184	188	122
LARGE	9 Lrg	SII4	120	120	186	188	122
LARGE	9 Lrg	SII4	116	120	184	188	120
LARGE	9 Lrg	SII4	120	120	188	188	122
LARGE	9 Lrg	SII4	116	120	184	188	120
LARGE	9 Lrg	SII4!	120	120	184	188	122
LARGE	9 Lrg	SII44	120	120	184	184	122
LARGE	9 Lrg	SII44	120	120	184	184	122
LARGE	9 Lrg	SII44	120	120	184	184	122
LARGE	9 Lrg	SII44	-9	-9	184	184	122
LARGE	9 Lrg	SII44	120	120	184	184	122
LARGE	9 Lrg	SII44	120	120	184	184	122
LARGE	9 Lrg	SII44	120	120	184	184	122
LARGE	9 Lrg	SII44	120	120	184	184	122
LARGE	9 Lrg	SII44	120	124	184	186	-9
LARGE	9 Lrg	SII44	120	120	184	184	122
LARGE	9 Lrg	SII44	120	120	184	184	122

LARGE	9 Lrg	SII44!	120	120	184	184	122
LARGE	9 Lrg	SII53	120	120	184	184	118
LARGE	9 Lrg	SII53	120	120	184	184	118
LARGE	9 Lrg	SII53	120	120	184	184	120
LARGE	9 Lrg	SII53	120	120	184	186	120
LARGE	9 Lrg	SII53	120	120	184	184	120
LARGE	9 Lrg	SII53	120	120	184	184	120
LARGE	9 Lrg	SII53!	120	120	184	184	120
LARGE	9 Lrg	SII54	120	124	184	184	120
LARGE	9 Lrg	SII54	116	120	184	186	120
LARGE	9 Lrg	SII54!	120	124	184	184	120
MEDIUM	7 Med	RC10	114	120	186	186	122
MEDIUM	7 Med	RC10	114	120	184	186	124
MEDIUM	7 Med	RC10	120	120	184	184	122
MEDIUM	7 Med	RC10	120	124	184	186	124
MEDIUM	7 Med	RC10	120	120	184	184	124
MEDIUM	7 Med	RC10	120	124	184	184	124
MEDIUM	7 Med	RC10	116	120	184	184	120
MEDIUM	7 Med	RC10	120	124	184	186	122
MEDIUM	7 Med	RC10	114	120	184	184	120
MEDIUM	7 Med	RC10	114	120	184	186	124
MEDIUM	7 Med	RC10!	114	120	184	186	122
MEDIUM	7 Med	RC11	120	124	184	184	120
MEDIUM	7 Med	RC11	114	120	184	184	120
MEDIUM	7 Med	RC11	116	120	184	184	126
MEDIUM	7 Med	RC11	116	124	184	186	120
MEDIUM	7 Med	RC11	116	120	184	184	120
MEDIUM	7 Med	RC11	120	124	184	184	126
MEDIUM	7 Med	RC11	116	120	184	184	120
MEDIUM	7 Med	RC11	120	124	184	184	120
MEDIUM	7 Med	RC11	116	124	184	186	-9
MEDIUM	7 Med	RC11!	116	120	184	184	120
MEDIUM	7 Med	RC13	120	124	186	186	118
MEDIUM	7 Med	RC13	120	124	186	186	122
MEDIUM	7 Med	RC13	120	124	184	186	122
MEDIUM	7 Med	RC13	120	124	186	186	122
MEDIUM	7 Med	RC13	120	120	184	184	122
MEDIUM	7 Med	RC13	120	120	184	184	122
MEDIUM	7 Med	RC13	120	124	184	186	122
MEDIUM	7 Med	RC13	120	120	186	186	122
MEDIUM	7 Med	RC13	120	124	184	186	122
MEDIUM	7 Med	RC13!	120	124	184	186	122
MEDIUM	7 Med	RC37	116	120	184	186	120
MEDIUM	7 Med	RC37	116	120	184	186	118
MEDIUM	7 Med	RC37	120	120	184	186	120
MEDIUM	7 Med	RC37	116	120	184	186	118
MEDIUM	7 Med	RC37	120	120	184	186	118
MEDIUM	7 Med	RC37	116	116	180	184	120
MEDIUM	7 Med	RC37	120	120	184	186	118

MEDIUM	7 Med	RC37	116	120	184	186	120
MEDIUM	7 Med	RC37!	116	120	184	186	118
MEDIUM	7 Med	RC40	120	124	184	184	118
MEDIUM	7 Med	RC40	120	124	184	186	120
MEDIUM	7 Med	RC40	120	124	184	184	118
MEDIUM	7 Med	RC40	120	120	184	184	118
MEDIUM	7 Med	RC40	120	124	184	186	118
MEDIUM	7 Med	RC40	120	124	184	186	118
MEDIUM	7 Med	RC40	120	120	184	186	120
MEDIUM	7 Med	RC40	116	124	184	184	118
MEDIUM	7 Med	RC40	120	124	184	186	118
MEDIUM	7 Med	RC40!	120	124	184	186	118
MEDIUM	7 Med	RC8	114	120	184	184	118
MEDIUM	7 Med	RC8	120	124	184	184	120
MEDIUM	7 Med	RC8	120	120	184	184	120
MEDIUM	7 Med	RC8	124	124	184	184	118
MEDIUM	7 Med	RC8	120	124	184	184	122
MEDIUM	7 Med	RC8!	120	124	184	184	120
MEDIUM	6 Med	Bu16!	120	124	-9	-9	116
MEDIUM	6 Med	Bu16	120	120	186	188	120
MEDIUM	6 Med	Bu16	120	124	186	188	120
MEDIUM	6 Med	Bu16	120	120	188	188	118
MEDIUM	6 Med	Bu16	120	120	188	188	116
MEDIUM	6 Med	Bu16	120	120	188	188	118
MEDIUM	6 Med	Bu16	120	120	186	188	116
MEDIUM	6 Med	Bu16	120	120	188	188	118
MEDIUM	6 Med	Bu17!	116	120	184	186	120
MEDIUM	6 Med	Bu17	120	120	184	186	120
MEDIUM	6 Med	Bu17	120	120	184	184	120
MEDIUM	6 Med	Bu17	120	120	184	184	120
MEDIUM	6 Med	Bu17	120	120	184	184	118
MEDIUM	6 Med	Bu17	120	120	184	186	118
MEDIUM	6 Med	Bu17	120	120	184	184	120
MEDIUM	6 Med	Bu17	120	124	184	186	118
MEDIUM	6 Med	Bu17	120	120	184	186	122
MEDIUM	6 Med	Bu17	120	120	184	186	120
MEDIUM	6 Med	Bu17	120	120	184	184	120
MEDIUM	6 Med	Bu19!	116	124	184	184	118
MEDIUM	6 Med	Bu19	116	124	184	184	118
MEDIUM	6 Med	Bu19	120	124	184	184	118
MEDIUM	6 Med	Bu19	120	124	184	184	118
MEDIUM	6 Med	Bu19	120	124	184	184	118
MEDIUM	6 Med	Bu19	120	124	184	186	118
MEDIUM	6 Med	Bu19	116	116	184	184	118
MEDIUM	6 Med	Bu19	116	124	184	184	118
MEDIUM	6 Med	Bu19	120	124	184	186	118
MEDIUM	6 Med	Bu19	124	124	184	184	120
MEDIUM	6 Med	Bu20!	116	120	184	186	120
MEDIUM	6 Med	Bu20	116	124	184	186	120

MEDIUM	6 Med	Bu20	116	120	184	186	120
MEDIUM	6 Med	Bu20	120	124	184	184	122
MEDIUM	6 Med	Bu20	120	120	186	186	118
MEDIUM	6 Med	Bu20	112	116	184	184	120
MEDIUM	6 Med	Bu20	112	116	184	184	120
MEDIUM	6 Med	Bu20	116	120	184	186	116
MEDIUM	6 Med	Bu20	116	120	186	186	118
MEDIUM	6 Med	BUI48!	116	120	184	188	120
MEDIUM	6 Med	Bu48	120	124	184	188	120
MEDIUM	6 Med	Bu48	120	120	184	184	118
MEDIUM	6 Med	Bu48	120	124	184	188	118
MEDIUM	6 Med	Bu48	116	120	188	188	118
MEDIUM	6 Med	Bu48	120	120	188	188	118
MEDIUM	6 Med	Bu48	120	124	184	188	118
MEDIUM	6 Med	Bu49!	120	124	184	186	-9
MEDIUM	6 Med	Bu49	116	124	184	184	120
MEDIUM	6 Med	Bu49	116	120	184	184	120
MEDIUM	6 Med	Bu49	116	120	184	188	122
MEDIUM	6 Med	Bu49	120	124	184	184	122
MEDIUM	6 Med	Bu49	116	120	184	186	122
MEDIUM	6 Med	Bu49	116	120	184	186	120
MEDIUM	6 Med	Bu49	116	124	186	188	120
MEDIUM	6 Med	Bu49	116	124	186	188	122
MEDIUM	6 Med	Bu55!	116	116	184	184	120
MEDIUM	6 Med	Bu55	116	116	184	188	120
MEDIUM	6 Med	Bu55	116	116	184	184	120
MEDIUM	6 Med	Bu60!	120	124	184	184	124
MEDIUM	6 Med	Bu60	120	124	184	186	122
MEDIUM	6 Med	Bu60	120	124	184	186	122
MEDIUM	6 Med	Bu60	120	120	184	184	122
MEDIUM	6 Med	Bu60	120	124	184	186	122
MEDIUM	6 Med	Bu60	120	120	184	184	122
MEDIUM	6 Med	Bu60	120	120	184	184	122
MEDIUM	6 Med	Bu60	120	124	184	186	122
MEDIUM	6 Med	Bu60	120	124	184	186	122
MEDIUM	6 Med	BU63!	120	120	184	184	120
MEDIUM	6 Med	BU63	120	120	184	184	120
MEDIUM	6 Med	BU63	120	120	184	188	120
MEDIUM	6 Med	BU63	120	124	184	188	120
MEDIUM	6 Med	BU63	120	124	184	188	120
MEDIUM	6 Med	BU70!	120	124	184	184	122
MEDIUM	6 Med	BU70	120	122	184	184	120
MEDIUM	6 Med	BU70	120	124	184	184	122
MEDIUM	6 Med	BU70	120	124	184	186	122
MEDIUM	6 Med	BU70	124	124	184	184	122
MEDIUM	6 Med	BU70	120	120	184	184	122
MEDIUM	6 Med	BU70	120	124	184	184	122
MEDIUM	6 Med	BU70	120	124	184	186	120
MEDIUM	6 Med	BU70	120	124	184	184	122

MEDIUM	6 Med	BU9!	116	120	186	186	-9
MEDIUM	6 Med	BU9	116	124	186	188	120
MEDIUM	6 Med	BU9	116	120	186	188	122
MEDIUM	6 Med	BU9	120	124	186	186	120
MEDIUM	6 Med	BU9	120	124	186	186	116
MEDIUM	6 Med	BU9	120	124	186	186	116
MEDIUM	6 Med	BU9	120	124	186	186	116
MEDIUM	6 Med	BU9	120	124	186	186	116
MEDIUM	6 Med	BU9	120	124	186	186	116
MEDIUM	6 Med	BU9	120	124	186	188	118
MEDIUM	6 Med	BU9	120	124	186	186	116

	Primer 4		Primer 5		Primer 6	
1233-2	4536-1	4536-2	4483-1	4483-2	1808-1	1808-2
122	183	193	220	220	300	300
122	188	193	206	220	300	300
124	193	193	220	230	300	300
122	193	193	220	230	297	300
122	193	193	206	214	297	300
124	193	193	214	220	297	300
122	193	193	214	220	297	300
-9	193	193	214	220	297	300
128	193	193	228	228	297	300
120	193	193	206	220	300	309
120	193	193	228	228	-9	-9
124	193	193	220	224	-9	-9
120	193	193	220	228	-9	-9
120	193	193	220	228	-9	-9
122	193	193	-9	-9	297	300
122	193	193	216	228	297	297
122	193	193	228	228	297	300
122	193	193	206	228	300	300
122	193	193	216	220	300	300
122	193	193	220	228	297	300
122	193	193	220	228	297	300
124	183	193	220	233	300	300
124	183	193	233	233	294	300
124	186	193	220	220	300	303
126	186	193	220	220	300	303
126	186	193	220	220	300	303
126	186	193	220	220	300	303
126	186	193	220	220	303	309
124	186	193	220	220	297	300
124	186	186	220	220	309	309
126	186	193	206	220	-9	-9
126	186	193	220	220	300	309
124	193	193	224	224	300	309
120	193	193	-9	-9	-9	-9
124	193	193	220	224	309	309
124	193	193	216	224	300	309
120	193	199	220	224	300	309
124	193	193	224	228	300	309
120	193	193	224	224	294	309
120	193	193	220	224	303	309
126	193	193	220	224	300	309
126	193	193	220	224	300	309
120	193	193	220	224	300	309
122	186	193	220	220	303	306
122	186	193	220	228	297	306
120	183	193	220	220	297	300
120	193	193	206	220	294	306

122	186	193	220	228	297	306
122	186	193	210	220	303	306
122	186	193	220	228	297	306
122	193	193	220	230	-9	-9
122	193	193	220	230	300	303
122	193	193	206	230	-9	-9
122	193	193	206	230	300	306
122	193	193	206	220	-9	-9
122	193	193	206	230	300	303
122	193	193	206	220	-9	-9
122	186	193	206	220	300	300
122	186	193	206	216	300	300
122	193	193	-9	-9	-9	-9
122	193	193	-9	-9	-9	-9
122	193	193	206	220	-9	-9
122	193	193	206	206	-9	-9
122	193	193	206	206	294	294
122	193	193	206	206	294	300
122	193	193	206	220	294	300
122	186	193	206	220	294	300
122	193	193	206	220	294	300
122	186	193	206	206	294	300
122	193	193	206	206	294	300
120	193	193	220	220	300	300
120	193	193	220	230	300	300
122	193	193	220	230	300	303
120	193	193	220	220	300	300
118	193	193	220	218	300	300
118	193	193	220	226	294	300
120	193	193	220	230	300	300
122	193	193	220	220	300	300
122	193	193	206	220	297	309
124	193	193	220	230	297	300
126	193	193	206	220	297	300
122	193	193	220	220	297	300
122	193	193	220	220	297	297
122	193	193	220	220	300	309
122	193	193	220	220	297	300
122	193	193	228	228	300	303
122	193	193	228	228	300	303
122	193	193	228	228	300	303
122	193	193	-9	-9	300	300
122	193	193	228	228	300	303
122	193	193	228	228	300	303
122	193	193	228	228	300	300
122	193	193	228	228	300	303
-9	193	193	228	228	-9	-9
122	193	193	228	228	300	303
122	193	193	228	228	300	303

122	193	193	228	228	300	303
122	193	193	220	230	300	309
120	193	193	220	220	300	309
122	193	193	220	220	300	303
122	193	193	220	220	300	309
122	193	193	220	220	300	309
122	193	193	220	220	300	300
122	193	193	220	220	300	309
122	193	193	206	206	-9	-9
122	193	193	230	230	294	294
122	193	193	206	230	294	300
122	193	193	220	220	300	300
126	193	193	220	220	-9	-9
122	193	193	220	220	300	300
126	193	193	220	220	294	300
126	193	193	220	220	294	300
126	193	193	220	224	300	300
122	187	193	220	226	300	300
124	193	193	220	220	300	300
124	193	193	220	220	300	300
124	193	193	220	220	294	300
124	193	193	220	220	300	300
120	193	193	220	220	-9	-9
120	193	193	206	220	300	300
126	193	193	220	222	-9	-9
120	193	193	220	220	-9	-9
126	193	193	220	220	-9	-9
126	193	193	220	220	294	300
126	193	193	220	220	-9	-9
120	193	193	220	220	294	300
-9	193	193	-9	-9	-9	-9
126	193	193	220	220	294	300
122	193	193	206	220	300	300
126	193	193	206	220	294	300
126	193	193	206	208	294	300
126	193	193	206	220	294	300
122	187	193	206	220	300	303
122	193	193	206	220	300	300
122	193	193	206	220	300	300
122	193	193	206	220	300	300
122	187	193	206	226	-9	-9
122	187	193	206	220	300	300
120	193	193	216	220	300	303
120	193	193	220	220	291	300
120	193	193	220	220	300	300
120	187	193	220	220	300	300
118	193	193	220	230	300	300
120	190	193	220	220	300	303
118	187	193	220	230	300	300

122	187	193	220	220	300	300
120	187	193	220	220	300	300
120	193	193	220	220	300	300
122	182	193	220	220	300	300
118	193	193	220	220	300	300
122	193	193	220	220	-9	-9
124	193	193	206	220	300	300
120	182	193	220	220	300	300
122	193	193	220	220	-9	-9
120	193	193	220	224	300	300
120	186	193	220	220	300	300
120	182	193	220	220	300	300
122	193	193	220	224	300	300
122	186	193	224	224	300	300
122	193	193	224	228	300	300
122	186	193	220	224	300	303
124	193	193	224	228	300	300
122	186	193	224	224	300	300
120	193	193	-9	-9	300	303
120	193	193	206	224	300	303
120	193	193	224	230	303	309
120	193	193	206	224	300	303
116	193	193	206	206	300	303
120	193	193	206	206	300	303
120	193	193	206	224	300	303
120	193	193	224	224	300	303
122	186	193	208	220	294	300
122	193	193	220	220	294	294
120	193	193	208	208	294	294
122	193	193	208	208	294	294
120	193	193	208	220	294	294
120	193	193	220	220	294	294
122	193	193	220	220	294	294
120	193	193	220	220	294	300
122	193	193	208	220	294	294
122	193	193	220	220	294	294
122	193	193	220	220	294	294
120	193	193	220	220	300	309
120	193	193	220	220	300	309
122	193	193	200	220	300	309
120	193	193	220	220	309	309
120	193	193	220	220	309	309
120	183	193	220	220	300	309
120	193	193	206	220	303	309
120	193	193	206	220	303	309
120	193	193	220	220	300	309
122	193	193	220	220	309	309
122	193	193	220	224	300	309
122	193	193	220	224	300	309

124	193	193	220	224	300	300
124	193	193	220	220	309	309
120	193	193	220	224	309	309
122	193	193	220	228	294	309
122	193	193	220	228	294	309
120	193	193	220	228	294	309
120	193	193	220	228	300	309
124	184	193	220	224	300	300
124	193	193	220	224	294	300
120	188	193	220	220	300	300
120	188	193	220	220	300	300
120	188	193	220	224	300	300
120	188	193	220	220	300	300
120	188	193	220	220	300	300
-9	186	193	220	226	294	300
124	193	193	220	226	294	300
124	193	193	226	226	294	300
124	193	193	220	226	300	300
124	193	193	220	226	294	300
124	193	193	220	226	294	300
124	193	193	220	224	294	300
124	193	193	224	226	294	294
124	193	193	224	226	294	294
120	188	193	226	226	300	300
124	188	193	226	226	300	300
120	188	193	226	226	300	300
124	188	193	206	206	300	309
124	193	193	200	206	300	300
124	186	193	206	220	309	309
124	186	193	206	206	300	309
124	193	193	200	206	300	309
124	193	193	206	220	-9	-9
124	188	193	206	220	-9	-9
124	188	193	206	220	309	309
124	188	193	206	220	300	309
120	193	193	220	220	300	300
122	193	193	220	220	300	300
122	193	193	220	224	300	303
122	193	193	220	220	300	300
120	184	193	206	220	300	309
124	193	193	224	224	300	300
124	193	193	224	224	294	300
124	186	193	224	224	300	300
124	193	193	224	228	300	300
124	193	193	224	224	300	300
124	193	193	224	224	300	300
124	193	193	224	224	300	300
122	188	193	208	224	294	300
124	193	193	224	230	300	300

-9	193	193	200	220	300	300
124	193	193	220	224	300	300
124	193	193	200	220	300	300
122	193	193	220	220	300	300
120	193	193	200	220	300	300
118	193	193	220	224	300	300
120	193	193	200	220	-9	-9
122	193	193	200	220	300	300
122	193	193	220	220	300	300
122	187	193	220	220	300	300
122	193	193	200	220	300	300
